# Apilimod alters TGFβ signaling pathway and prevents cardiac fibrotic remodeling

**DOI:** 10.7150/thno.55821

**Published:** 2021-04-19

**Authors:** Mathieu Cinato, Laurie Guitou, Amira Saidi, Andrei Timotin, Erwan Sperazza, Thibaut Duparc, Sergey N. Zolov, Sai Srinivas Panapakkam Giridharan, Lois S. Weisman, Laurent O. Martinez, Jerome Roncalli, Oksana Kunduzova, Helene Tronchere, Frederic Boal

**Affiliations:** 1 INSERM U1297 I2MC, Toulouse, France and Université Paul Sabatier, Toulouse, France.; 2 Life Sciences Institute, University of Michigan, Ann Arbor, USA.; 3 Department of Cardiology, Toulouse University Hospital, Toulouse, France.

**Keywords:** Heart failure, fibrotic remodeling, PIKfyve, Apilimod, TGFβ.

## Abstract

**Rationale:** TGFβ signaling pathway controls tissue fibrotic remodeling, a hallmark in many diseases leading to organ injury and failure. In this study, we address the role of Apilimod, a pharmacological inhibitor of the lipid kinase PIKfyve, in the regulation of cardiac pathological fibrotic remodeling and TGFβ signaling pathway.

**Methods:** The effects of Apilimod treatment on myocardial fibrosis, hypertrophy and cardiac function were assessed *in vivo* in a mouse model of pressure overload-induced heart failure. Primary cardiac fibroblasts and HeLa cells treated with Apilimod as well as genetic mutation of PIKfyve in mouse embryonic fibroblasts were used as cell models.

**Results:** When administered *in vivo*, Apilimod reduced myocardial interstitial fibrosis development and prevented left ventricular dysfunction. *In vitro*, Apilimod controlled TGFβ-dependent activation of primary murine cardiac fibroblasts. Mechanistically, both Apilimod and genetic mutation of PIKfyve induced TGFβ receptor blockade in intracellular vesicles, negatively modulating its downstream signaling pathway and ultimately dampening TGFβ response.

**Conclusions:** Altogether, our findings propose a novel function for PIKfyve in the control of myocardial fibrotic remodeling and the TGFβ signaling pathway, therefore opening the way to new therapeutic perspectives to prevent adverse fibrotic remodeling using Apilimod treatment.

## Introduction

Fibrosis is a conserved end-stage hallmark of a broad range of organ injuries and failure such as cardiac, kidney and pulmonary diseases 1. To date, there are limited therapeutic treatments available to limit fibrotic remodeling progression, largely due to the heterogeneous evolution of fibrosis in different pathological stimuli and organs. For instance, myocardial fibrotic remodeling is a common pathological process in heart diseases and a determinant step of development and progression of heart failure, thereby participating to cardiac diastolic and systolic dysfunctions 2. Fibrotic remodeling in the pathological heart is a highly orchestrated process in which cardiac fibroblasts are central cellular mediators 3. Activated cardiac fibroblasts have increased proliferative, migrative and invasive capacities. They are characterized by α-SMA contractile fibres and excessive production and deposition of extracellular matrix (ECM) proteins, e.g. collagen type I and III 3. Although TGFβ is recognized as one of the key mediators of fibroblast to myofibroblast transition, both *in vitro* and *in vivo*, the molecular control of its signaling and downstream effectors is still poorly understood 4. To date, TGFβ-based anti-fibrotic therapies are limited and new strategies to target its downstream signaling are needed. The TGFβ signaling pathway is diverse and tightly regulated. At the molecular level, TGFβ receptor type 2 (TGFβ-R2) binds directly to the TGFβ, associates with the TGFβ receptor type 1 (TGFβ-R1) and induces its phosphorylation on serine and threonine residues 5. This triggers the canonical signaling pathway through the Smad proteins, or the non-canonical pathways. At the plasma membrane, the TGFβ receptors have a rapid turnover, most likely linked to their internalization by endocytosis in a clathrin- or caveolin-dependent manner, determining the receptor fate and subsequent signaling 6, 7. Once internalized to an early endosomal EEA1-positive compartment, the receptors can be either recycled back to the plasma membrane or directed to the late endosomal CD63-positive compartment, ultimately leading to their lysosomal degradation 8.

PIKfyve is an evolutionarily conserved phosphoinositide 5-kinase that generates PI(3,5)P_2_ and is responsible of most of the cellular pool of PI5P 9,10. PIKfyve is a master regulator of membrane trafficking and signaling, mostly within the endosomal/endocytic system, where it controls the maturation of early endosomes to late endosomes/lysosomes through the conversion of PI3P into PI(3,5)P_2_ 9. Indeed, inhibition of PIKfyve by overexpression of a dominant-negative mutant 11 or via its pharmacological inhibition 12 alters endomembrane homoeostasis, leading to extensive endosomal vacuolation. *In vivo*, the functions of PIKfyve have begun to be unraveled using genetically engineered mice or pharmacological manipulation. PIKfyve-null mice are embryonically lethal 13, but hypomorphic PIKfyve^β-geo/β-geo^ mice, which expressed residual PIKfyve activity are viable and develop defects within multiple organs, such as in the nervous, cardiopulmonary and renal systems 14. Recently, a new potent and highly selective inhibitor of the kinase, Apilimod, has been identified through a high-throughput IL-12 inhibitor screening 15, 16. Apilimod binds to the catalytic C-terminal domain of PIKfyve, most likely to the ATP binding pocket 15. Recently, we took advantage of this inhibitor to unravel a novel role for PIKfyve in cardiomyocyte mitochondrial integrity, ROS production and apoptosis in response to stress, culminating in improved cardiac functions in a mouse model of diabetic cardiomyopathy 17. Additionally, we observed a reduction in myocardial fibrosis following chronic PIKfyve inhibition, suggesting a potential role of PIKfyve in cardiac fibroblasts reprogramming. Here, we now demonstrate that PIKfyve is required for the fibrotic remodeling of cardiac tissue after chronic myocardial injury. We provide the first evidence that pharmacological inhibition of PIKfyve by Apilimod prevents excessive fibrotic development through the dampening of the TGFβ/Smad signaling pathway and ultimately improves cardiac function. Altogether, these results unveil PIKfyve as a novel regulator of fibroblast activation and propose Apilimod as a promising anti-fibrotic molecular therapy.

## Methods

### Reagents and antibodies

Antibodies used in this study are: anti-Hsp90 (sc-13119), anti-GAPDH (sc-32233) and anti-Smad2/3 (sc-133098) from Santa Cruz Biotechnology; anti-αSMA (A5228, for western-blot) and anti-flag M2 from Sigma; anti-CD68 (GeneTex GTX41864), anti-GM130 (#610822) from BD Biosciences; anti-EEA1 (GTX109638) and anti-caveolin-1 (GTX100205) from GeneTex; anti-CD63 (MCA2142) was from AbD Serotec; anti-EGFR (D38B1), anti-phospho-Smad2 (#3108) and 3 (#9520), anti-αSMA (#19245, for immunofluorescence), anti-COL1 (#91144) from Cell Signaling Technologies; anti-PI(3,5)P_2_ (Z-P035) from Echelon; anti-HA.11 (16B12, Covance). Fluorescent Alexa-coupled secondary antibodies and DAPI were from Life Technologies and HRP-coupled secondary antibodies from Cell Signaling Technology. Apilimod was purchased from Axon Medchem. The plasmids encoding GFP-ICAM1 and P2Y12-GFP were as described 18. All other chemicals were from Sigma unless otherwise stated.

### Generation of the 6X-HA-PIKfyve plasmid

6xHA PIKfyve was generated with a cDNA sequence that that is 100% identical to GenBank sequence AAR19397. Due to the large size of the cDNA (6.2 kb), PIKfyve was cloned into pCMV-HA vector (635690, Clontech Laboratories, Inc) in two stages. Citrine-PIKfyve 19 was digested with BsrGI, the internal fragment (nucleotides 2510-6213; 3703 bp) was isolated to reinsert later. The remaining plasmid corresponding to the N- and C-terminal regions of PIKfyve, was ligated and this deletion construct of PIKfyve was PCR amplified using primers: forward, acgcgtcgaccatggccacagatgataagacg and reverse: aaggaaaaaagcggccgctcagcaattcagacccaagc. The PCR fragment and pHA-CMV vector were cleaved with SalI and NotI and the PIKfyve deletion construct was inserted into the vector. A resultant clone with the correct sequence was further digested with BsrGI. The internal BsrGI digested fragment was re-ligated back to generate 1xHA-PIKfyve. This construct was further digested with SfII and SalI and an additional 5XHA -tag (gctcttatggccatggaggccTACCCATACGATGTTCCTGACTATGCGGGCTATCCCTATGACGTCCCGGACTATGCAGGATCCTATCCATATGACGTTCCAGATTACGCTtcaTACCCTTATGACGTGCCCGATTACGCCGGCagtTACCCTTACGATGTCCCAGATTACGCTCCGcggtcgaccatggccaca encoding 5xHA tag: YPYDVPDYA G YPYDVPDYA GS YPYDVPDYA S YPYDVPDYA GS YPYDVPDYA) was inserted at the N-terminus of PIKfyve using Gibson assembly to generate a 6xHA-PIKfyve wild type construct.

### Animal studies

Two-month old wild-type male C57BL6/J mice were purchased from Envigo. Transverse aortic constriction (TAC) was produced by constriction of the ascending aorta around a 26-gauge needle with the use of a 7-0 prolene suture, maintained for 4 weeks as described previously 20. Sham-operated mice underwent a similar procedure without constriction of the ascending aorta. Animals were randomly divided into four groups: (i) Sham/vehicle (n = 7), (ii) Sham/Apilimod (n = 6), (iii) TAC/vehicle (n = 7), and (iv) TAC/Apilimod (n = 9). Mice were treated 4 days after surgery and then every day for 4 weeks with Apilimod (2 mg/kg/day, i.p.) or vehicle (DMSO), corresponding to a final DMSO concentration of 50% diluted in PBS as previously described 17. For acute Apilimod treatment, mice were treated intraperitoneally with Apilimod (2 mg/kg/day) for 8 consecutive days. Cardiac fibroblasts were isolated as described below, let to adhere on gelatin-coated coverslips for 4 h, and process further for cell surface binding of fluorescent TGFβ. For the washout experiments, cells were extensively washed and further cultured for 24-48 h before being processed.

### Echocardiography

Blinded echocardiography was performed as described 17 on isoflurane-anesthetized mice using a Vivid7 imaging system (General Electric Healthcare) equipped with a 14-MHz sectorial probe. Two-dimensional images were recorded in parasternal long- and short-axis projections, with guided M-mode recordings at the midventricular level in both views. Left ventricular (LV) dimensions and wall thickness were measured in at least five beats from each projection and averaged. Shortening fraction (SF) and ejection fraction (EF) were calculated from the two-dimensional images.

### Morphology

Hematoxylin-eosin, Sirius red and fluorescent WGA stainings on 10 μm heart cryosections were performed according to standard methods. The extent of cardiac fibrosis was quantified using ImageJ software 17, 20. Quantification of myocyte cross-sectional area was performed as described 17.

### Cell culture, transfection and treatments

Mice primary cardiac fibroblasts were isolated and cultured according to the method described previously 20. Briefly, the heart was isolated from adult male C57BL6/J mice, triturated and was then incubated with Digestion Buffer [HBSS (Sigma H8264); BSA (1 mg/mL); Pancreatin NB (Serva #31442; 0.5 mg/mL) and Collagenase NB4 (Serva #17454.01; 0.1 mg/mL)] at 37°C for four consecutive rounds of digestion. The suspension was centrifuged at 300 g for 5 min and the cell pellet was resuspended in DMEM F12 medium (Sigma D6434) supplemented with 10% FBS, penicillin (20 U/mL)-streptomycin (20 µg/mL) and 2 mM glutamine and cultured in a 37°C, 5% CO_2_ incubator. Primary cardiac fibroblasts were used up to passage 3. HeLa, HEK and MEF cells from hypomorphic PIKfyve^β-geo/β-geo^ mice were cultured in DMEM supplemented with 10% FBS, penicillin (20 U/mL)-streptomycin (20 µg/mL). For TGFβ treatment, the cells were pretreated for 30 min with Apilimod (100 nM) or DMSO (vehicle only) and then subjected to TGFβ stimulation (10 ng/mL) for 48 h unless otherwise stated. Cardiac fibroblasts, HeLa and HEK cells were transiently transfected using JetPRIME (Polyplus transfection), X-tremeGENE 9 (Roche) or TransIT-X2 (Mirus) according to manufacturers' instructions. After 24 h, the cells were serum-starved and treated with Apilimod 100 nM for 16 h. Cells were fixed with PFA, immunofluorescence was performed as described 21 and imaged on a Zeiss LSM-780 or a LSM900 confocal microscope. For PI5P staining, the previously described biotinylated PHD probe was used 22 in combination with a streptavidin-Alexa546. Colocalization studies were performed using ImageJ. The quantification of TGFβ-R2 internalization was essentially done as described 18. Briefly, an outer ring just surrounding the cell was drawn, which yielded the total fluorescence intensity, i.e., the total amount of TGFβ-R2 expressed in the cell. An inner ring just below the plasma membrane was then drawn, which yielded the amount of TGFβ-R2 internalized into the cell. The ratio between the two values gave the amount of internalized TGFβ-R2, normalized against the total amount of receptor expressed in the cell.

### Cell-surface binding of fluorescent TGFβ

TGFβ was fluorescently labeled using the AlexaFluor-555 protein Labeling Kit (A20174, Invitrogen) according to manufacturer's instructions. For cell-surface binding of fluorescent TGFβ, cells were washed with HBSS at 4°C and incubated with A555-TGFβ (500ng/mL) in HBSS at 4°C for 30min. Cells were extensively washed and processed further for immunofluorescence.

### Cell migration, polarization and invasion assays

Cell migration was assessed using the wound healing assay. Briefly, a confluent fibroblast culture was scratched using a micropipet tip. Twenty-two hours later, cells were fixed and nuclei were stained with DAPI. The wound closure was measured using ImageJ. Cell polarization was assessed as described 23. Briefly, fixed cells were stained using fluorescent phalloidin (to stain actin cytoskeleton) and an anti-GM130 antibody (to stain the Golgi apparatus). A cell was considered polarized if the Golgi apparatus was oriented within a 120° angle facing the wound. Cell invasion assay was performed in matrigel coated modified-Boyden chamber assay and assessed by spectrophotometric measurements as described 24.

### Collagen gel contraction assay

Neonatal rat cardiac fibroblasts were isolated essentially as described 25, plated on gelatin-coated dishes and used at maximum passage 1. Collagen gel contraction assay was performed as described 26. Briefly, cardiac fibroblasts were seeded in a collagen gel, treated, and the contraction of the gel was assessed after 72 h. After PFA-fixation, the gels were imaged on an Axio Observer Z1 microscope (Zeiss) using the mosaic function. The area of the gel was measured and normalized against the total well area.

### Protein extraction and western blotting

Proteins from cardiac tissues or cells were extracted using RIPA buffer and quantified using the Bio‐Rad Protein Assay (Bio‐Rad). Proteins were loaded in Laemmli sample buffer, denaturated at 70°C for 15 min, and resolved by SDS-PAGE and Western blotting. Immunoreactive bands were detected by chemiluminescence with the Clarity Western ECL Substrate (Bio‐Rad) on a ChemiDoc MP Acquisition system (Bio‐Rad).

### RNA Extraction and Quantitative Polymerase Chain Reaction (qRT-PCR)

Total RNAs were isolated from mice primary fibroblasts or tissues using the RNeasy mini kit (Qiagen) or the Direct-zol DNA/RNA miniprep kit (Zymo Research). Total RNAs were reverse transcribed using Superscript II reverse transcriptase (Invitrogen) or M-MLV Reverse Transcriptase (Promega) in the presence of a random hexamers. Real-time quantitative PCR was performed as previously described 27 using the MESA BLUE qPCR MasterMix Plus (Eurogentec) or the SsoFast™ EvaGreen® Supermix (Bio-Rad). The expression of target mRNA was normalized against house-keeping genes (Gapdh, Rps29, Rplpo or Hprt) mRNA expression. Primers used in this study are detailed in supplementary Table S1.

### Data and Statistical analysis

The data and statistical analysis comply with the recommendations on experimental design and analysis in pharmacology 28. Data are expressed as mean ± SEM. Comparison between two groups was performed by Student's two-tailed t-test while comparison of multiple groups was performed by one-way ANOVA followed by a Bonferroni's post hoc test using GraphPad Prism version 9.0.2 (GraphPad Software, Inc).

### Study approval

The investigation conforms to the ARRIVE guidelines, the Guide for the Care and Use of Laboratory Animals published by the US National Institutes of Health (NIH Publication No. 85-23, revised 1985) and was performed in accordance with the recommendations of the French Accreditation of the Laboratory Animal Care (approved by the local ethics committee).

## Results

### Apilimod protects against myocardial interstitial fibrosis development and prevents pressure overload-induced cardiac dysfunction in mice

**Figure 1:**
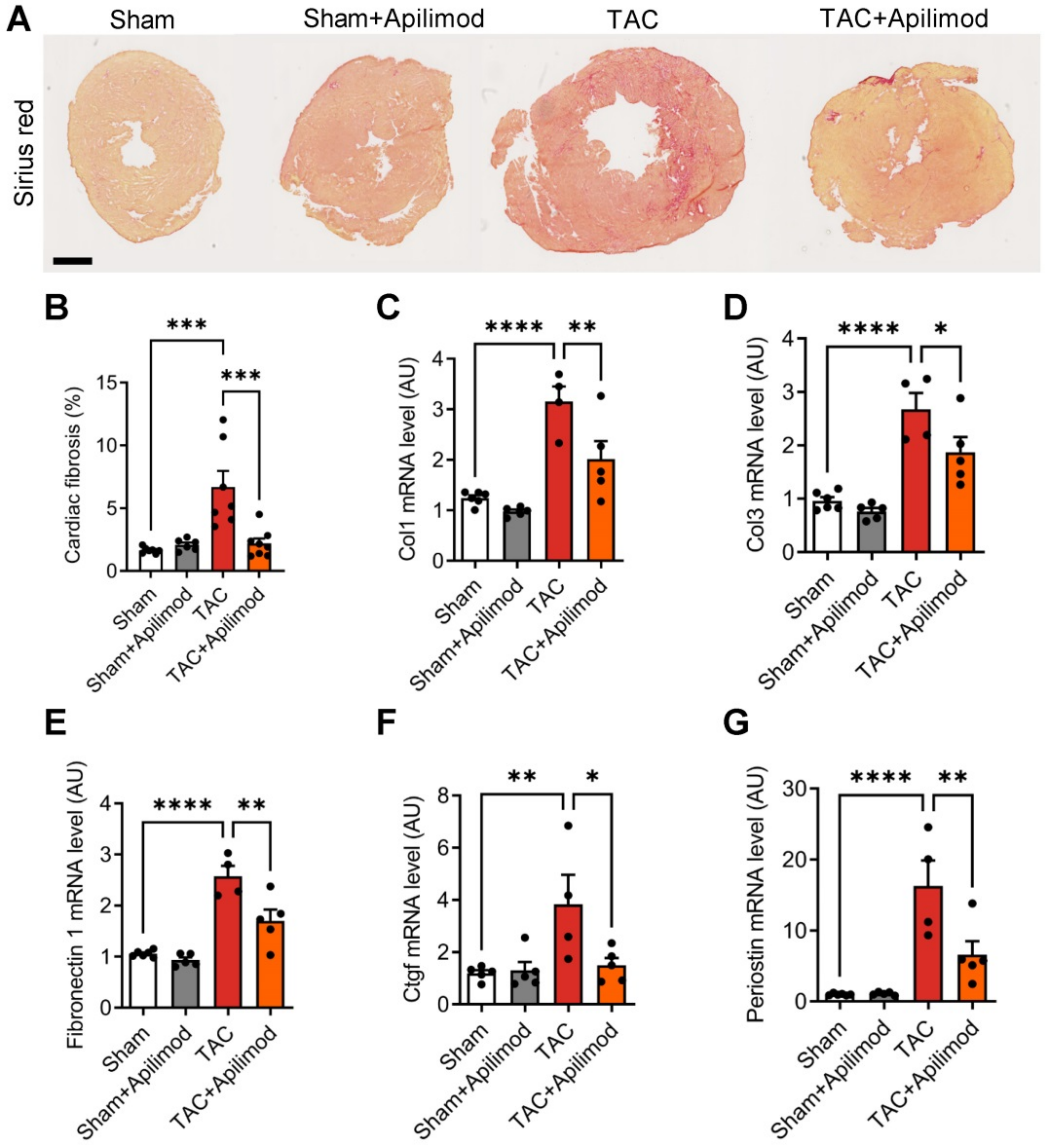
** Apilimod prevents cardiac fibrosis development in mice. (A)** Cardiac fibrosis was assessed on heart cryosections stained with Sirius red from mice sham-operated or subjected to TAC surgery treated intraperitoneally with Apilimod. Bar is 1 mm. **(B)** Quantification of cardiac fibrosis from (A). n = 6-8 mice per group. **(C-G)** Expression levels of myocardial collagen 1 (Col1, C), collagen 3 (Col3, D), fibronectin-1 (E), Ctgf (F) and periostin (G) by qRT-PCR. n = 6-8 mice per group. ANOVA followed by Bonferroni's post-hoc test, *p < 0.05; **p < 0.01; ***p < 0.001; ****p < 0.0001.

**Figure 2:**
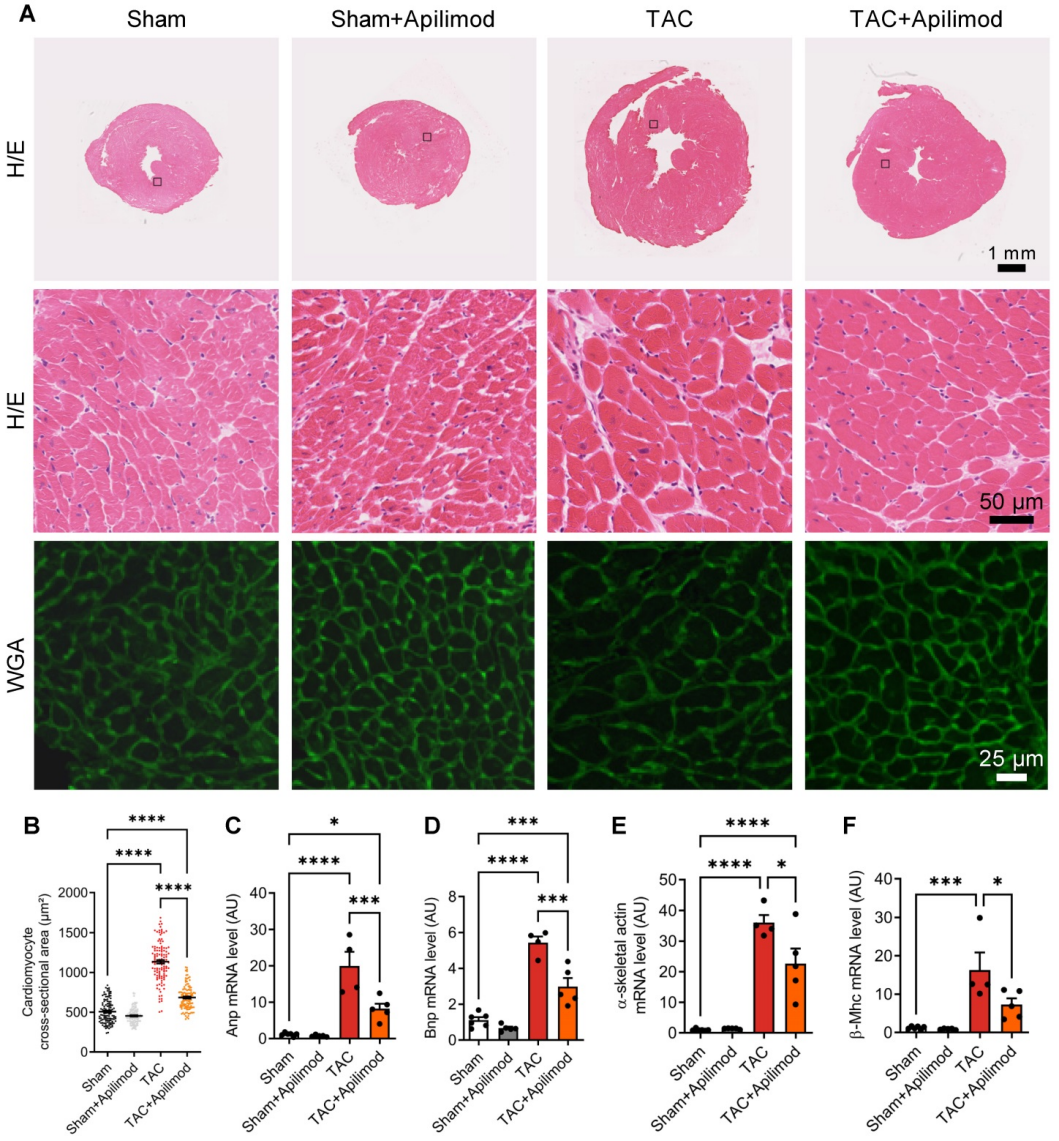
** Apilimod blunts cardiac hypertrophic response in TAC-mice. (A)** Heart cryosections of mice treated as indicated were stained with hematoxylin-eosin (H/E) or fluorescent WGA. **(B)** Quantification of cardiomyocytes cross-sectional area measured from (A). Quantification was performed from 3-4 images across 3-5 mice per group. **(C-F)** Expression levels of Anp, Bnp, α-skeletal actin and β-Mhc were measured by qRT-PCR in cardiac tissues. n = 4-6 mice per group. ANOVA followed by Bonferroni's post-hoc test, *p < 0.05; ***p < 0.001; ****p < 0.0001.

To address the effects of Apilimod in cardiac fibrotic remodeling, we used a mouse model of transverse aortic constriction (TAC). Cardiac pressure overload induced by four weeks TAC triggered a massive myocardial deposition of ECM proteins as shown by Sirius red staining, typical for end-stage fibrotic tissue remodeling (Figure 1A-B). Strikingly, Apilimod abrogated myocardial collagen fibre accumulation in TAC-mice (Figure 1A-B). Consistently, daily Apilimod treatment attenuated TAC-induced overexpression of cardiac pro-fibrotic genes including collagen 1 (Col1, Figure 1C), collagen 3 (Col3, Figure 1D), fibronectin 1 (Figure 1E) and connective tissue growth factor (Ctgf, Figure 1F). Moreover, upon Apilimod treatment, TAC-induced expression of periostin, a well-known marker of activated cardiac fibroblasts 29, was strongly inhibited (Figure 1G), suggesting that Apilimod prevents fibroblast activation *in vivo*. Consistently, TAC-stressed mice treated with Apilimod showed reduced level of α-SMA as demonstrated by immunofluorescent staining (Figure S1A). Moreover, we found that Apilimod decreased the recruitment of CD-68-positive macrophages (Figure S1B) and the production of the myocardial pro-inflammatory cytokines Il-6 (Figure S1C), Tnf-α (Figure S1D) and Ccl2 (Figure S1E). In addition, we found that Apilimod reduced cardiac hypertrophy induced by TAC, as shown morphologically (Figure 2A-B) and by the expression of the hypertrophic markers Anp (Figure 2C), Bnp (Figure 2D), α-skeletal actin (Figure 2E) and β-Mhc (Figure 2F). Consistently, echocardiography analysis showed that Apilimod reduced end-diastolic ventricular wall thickness, intraventricular septum thickness and left ventricular mass (Table 1) in TAC-stressed mice hearts. It has to be noted that Apilimod-treatment does not completely abrogate the hypertrophic response following TAC. Importantly, the myocardial anti-fibrotic effect of Apilimod culminated in the preservation of cardiac function in TAC-mice, as shown by improved EF and shortening fraction (Table 1 and Figure S1F, EF and SF respectively). These results suggest that Apilimod is a potent inhibitor of fibroblast activation and myocardial fibrosis development *in vivo*, leading to preserved cardiac performance following TAC surgery.

### Apilimod reduces cardiac fibroblast activation and differentiation *in vitro*

**Figure 3:**
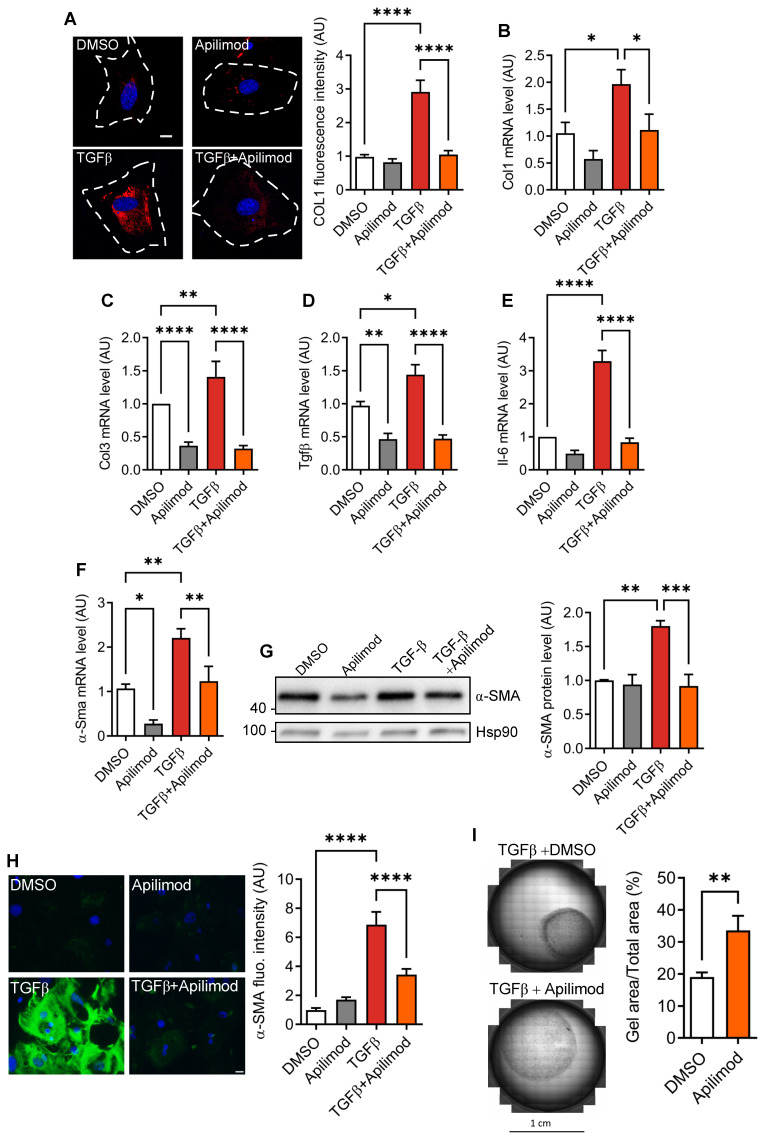
** Apilimod inhibits cardiac fibroblast activation. (A)** Cardiac fibroblasts were treated with TGFβ in the presence of Apilimod or DMSO, fixed and stained with an anti-collagen 1 antibody (shown in red). Nuclei were stained with DAPI (in blue). Bar is 10 µm. Quantification if shown on the right, n = 41-82 cells across 3 independent experiments. **(B-E)** Expression levels of collagen 1 (Col1, B), collagen 3 (Col3, C), Tgfβ (D) and Il-6 (E) were assessed by qRT-PCR on treated cardiac fibroblasts. **(F-H)** Expression level of α-Sma was assessed by qRT-PCR (F), western-blot (G) and immunofluorescence (H) on treated cardiac fibroblasts. **(I)** Collagen gel contraction assay on TGFβ activated cardiac fibroblasts treated with Apilimod or DMSO for 72 h. n = 4-6 independent experiments. ANOVA followed by Bonferroni's post-hoc test or Student's t-test (unpaired, two-tailed), *p < 0.05; **p < 0.01; ***p < 0.001, ****p < 0.0001.

Fibroblast activation can be induced *in vitro* by TGFβ treatment 20. After 48 h stimulation, we observed an increase in extracellular collagen fibre deposition as shown by collagen 1 immunostaining (Figure 3A). This was consistent with increased mRNA levels of pro-fibrotic factors collagen 1, collagen 3, Tgfβ (Figure 3B-D) and inflammatory cytokine Il-6 (Figure 3E). Strikingly, concomitant treatment of fibroblasts with Apilimod almost completely abrogated collagen accumulation (Figure 3A), pro-fibrotic (Figure 3B-D) and inflammatory Il-6 (Figure 3E) transcript levels. Next, we investigated the effect of Apilimod on the expression of the myofibroblast marker α-SMA. Apilimod treatment decreased both α-Sma mRNA (Figure 3F) and protein levels measured by western-blot and immunefluorescent staining (Figure 3G-H) in TGFβ-treated cardiac fibroblasts. Additionally, Apilimod markedly reduced the acquisition of the contractile phenotype of TGFβ stimulated cardiac myofibroblasts in a three-dimensional collagen gel contraction assay (Figure 3I).

**Figure 4:**
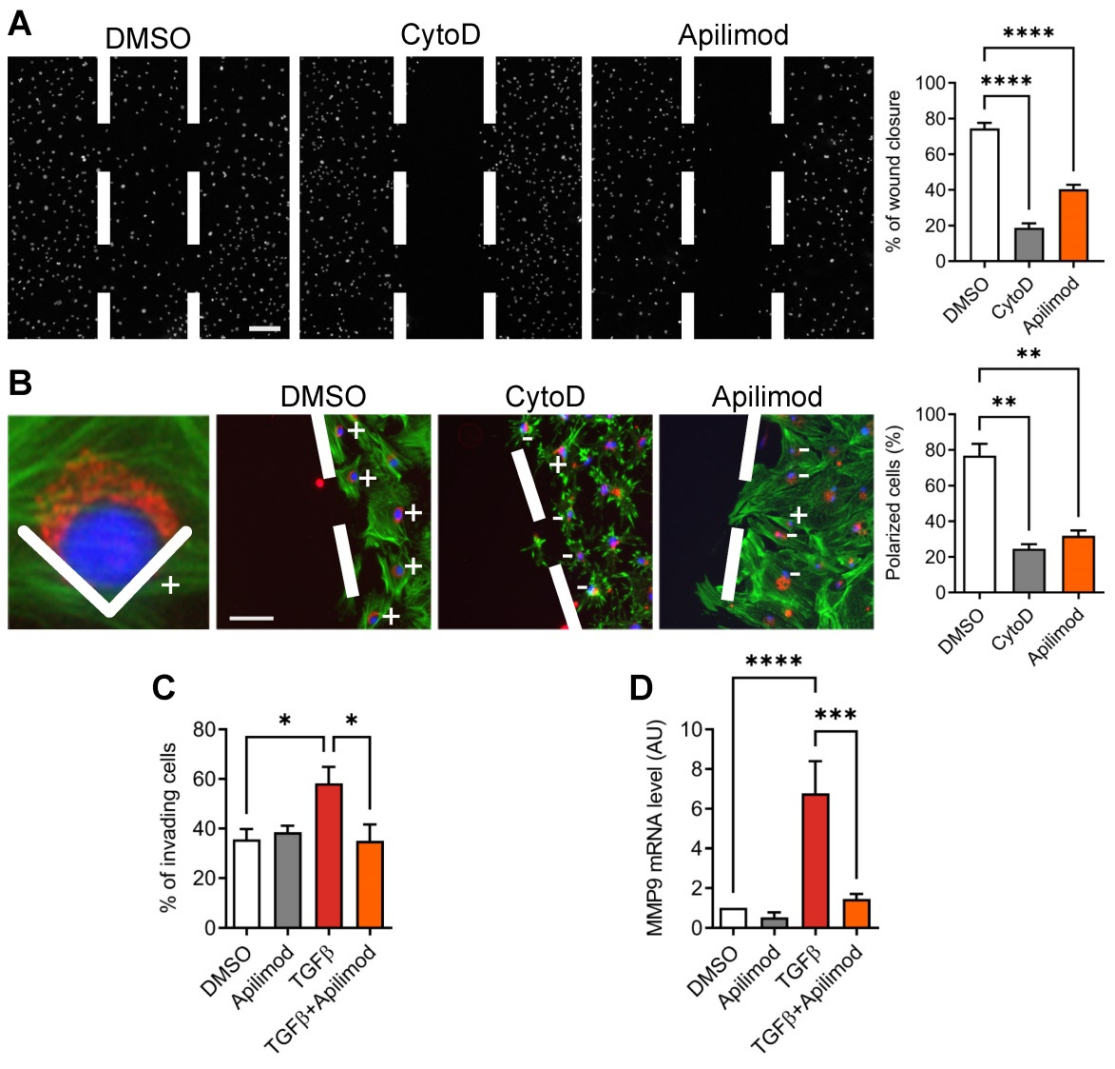
** Apilimod reduces cardiac fibroblasts migration and invasion. (A)** Migrative properties of treated cardiac fibroblasts were assessed using the wound-healing assay. Nuclei were stained with DAPI (shown in white). Bar is 200 µm. Results are from 3 independent experiments. **(B)** Cell polarity was scored by the positioning of the Golgi apparatus (stained with GM130, shown in red) towards the migration front. Phalloidin staining of the actin cytoskeleton was used as a counterstain (shown in green). Cell nuclei were stained with DAPI (in blue). Bar is 100 µm. Results are from n = 19-43 cells across 3 independent experiments. **(C)** Cardiac fibroblasts invasive properties were assessed using a modified Boyden chamber invasion assay. Results are from 3 independent experiments. **(D)** Expression level of Mmp9 was measured by qRT-PCR in treated cardiac fibroblasts. Results are from 3 independent experiments. ANOVA followed by Bonferroni's post-hoc test, *p < 0.05; **p < 0.01; ***p < 0.001; ****p < 0.0001.

The activation of cardiac fibroblasts is characterized by a migratory and invasive phenotype. Therefore, we looked at the effect of Apilimod on the migrative properties of TGFβ stimulated cardiac fibroblasts. Apilimod treatment abolished TGFβ-induced cell migration in a 2D wound-healing assay, as potently as cytochalasin-D, an inhibitor of actin cytoskeleton and cell migration (Figure 4A). These data indicate that Apilimod inhibits directed cell migration of cardiac fibroblasts. Cell polarization is a prerequisite to cell migration and can be monitored by the positioning of the Golgi apparatus between the nucleus and the leading edge of the cell toward the wounded area. As shown in Figure 4B, while in DMSO-treated cells most of the fibroblasts became polarized towards the wounded area, Apilimod completely abrogated this phenomenon, similarly to cytochalasin D treatment. Next, we investigated the effect of Apilimod on activated fibroblasts invasive properties in a more physiologically relevant model, i.e. a matrigel-coated modified Boyden chamber assay. As shown in Figure 4C, while TGFβ enhanced the number of invading cells, Apilimod treatment dramatically reduced TGFβ-stimulated invasion of primary cardiac fibroblasts. Consistently, we observed a down-regulation of the matrix metalloproteinase Mmp9, a key ECM remodeling enzyme, in Apilimod-treated TGFβ-stimulated fibroblasts (Figure 4D). Collectively, these results identify Apilimod as a potent inhibitor of cardiac fibroblast activation in response to TGFβ.

### Apilimod inhibits cardiac fibroblast activation through vacuolar sequestration of TGFβ-R2

**Figure 5:**
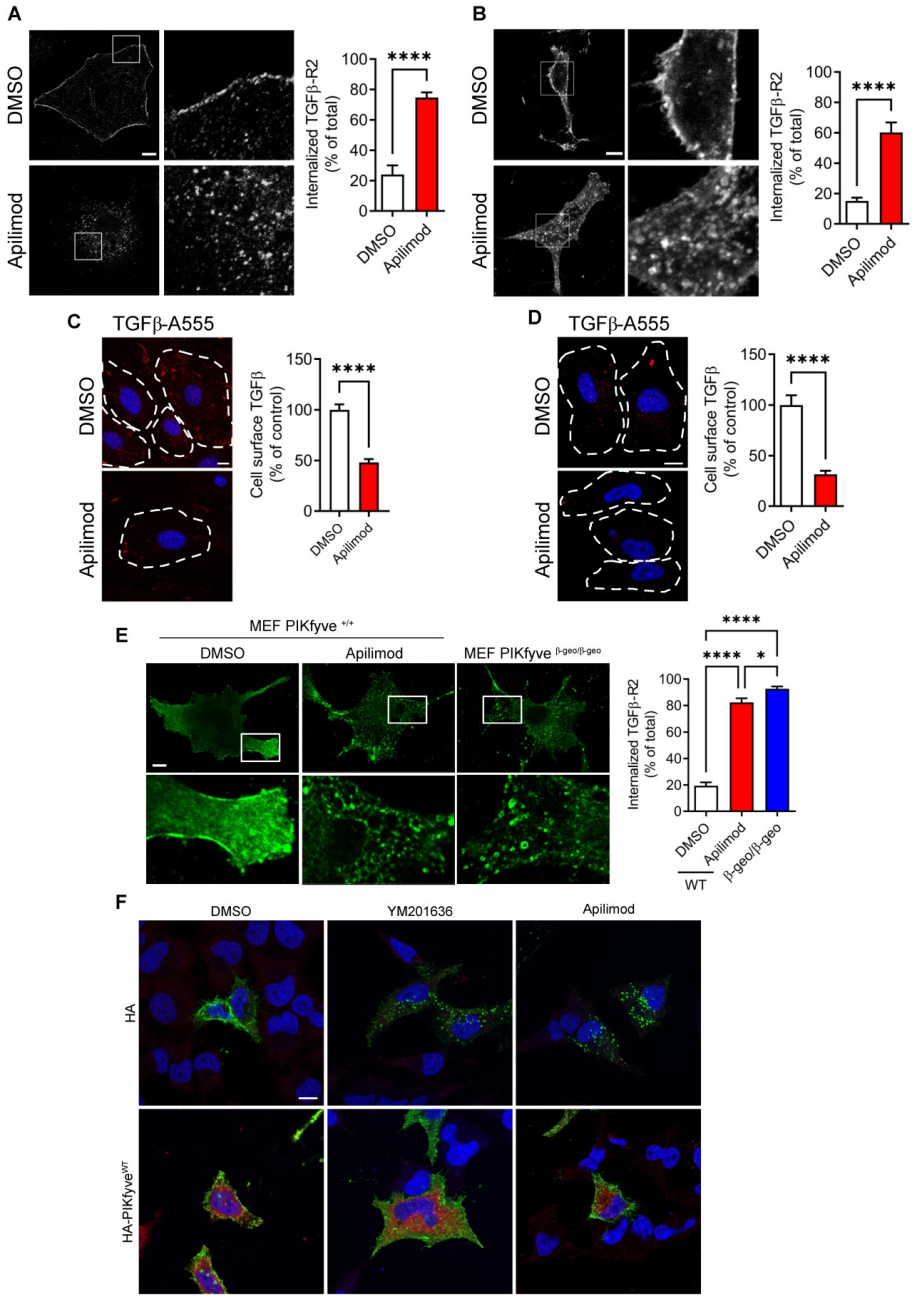
** PIKfyve inactivation triggers steady-state TGFβ-R2 internalization. (A-B)** Isolated primary cardiac fibroblasts (A) or HeLa cells (B) expressing flag-TGFβ-R2 were serum starved and treated with Apilimod or DMSO as indicated. Cells were fixed and stained with an anti-flag antibody. Bar is 10 µm. Results are from n = 9-16 cells across 3 independent experiments. **(C-D)** Cell surface binding of fluorescent TGFβ (TGFβ-A555, shown in red) on treated- cultured primary cardiac fibroblasts (C) or HeLa cells (D). Nuclei were stained with DAPI (shown in blue). Bar is 10 µm. n = 43-110 cells across 3 independent experiments. Student's t-test (unpaired, two-tailed), ****p < 0.0001.** (E)** Mouse embryonic fibroblasts (MEF) isolated from PIKfyve^βgeo/βgeo^ mice or wild-type littermates were transfected to express flag-TGFβ-R2, serum-starved and treated with Apilimod as indicated. Cells were fixed and stained with a flag antibody (green) and imaged by confocal microscopy. Bar is 10 µm. Right panel shows the quantification of flag-TGFβ-R2 internalization. n = 4-8 cells across 3 independent experiments. ANOVA followed by Bonferroni's post-hoc test or Student's t-test (unpaired, two-tailed), *p < 0.05; ****p < 0.0001. (**F**) HeLa cells were transfected with plasmids encoding flag-TGFβ-R2 and empty HA or HA-PIKfyve^WT^ vector as indicated. Cells were treated either with DMSO, YM201636 or Apilimod (100 nM) for 48 h, fixed and stained with anti-flag (shown in green) and anti-HA (in red) antibodies. Nuclei were stained with DAPI (in blue). Bar is 10 µm.

To gain further insight into the molecular mechanisms involved, we examined whether Apilimod was affecting TGFβ signaling pathway. After binding the TGFβ ligand, TGFβ receptor type 2 (TGFβ-R2) associates with TGFβ-R1, and the resulting complex is internalized to the endosomal compartment, as a requisite for its signaling 30. As shown in Figure 5A, in resting control cardiac fibroblasts, the TGFβ-R2 was localized at the plasma membrane, as expected for the endogenous receptor. Strikingly, we found that Apilimod massively induced TGFβ-R2 receptor relocalization into intracellular vesicles independently from any TGFβ stimulation (Figure 5A). This intracellular retention of the TGFβ-R2 was also observed in HeLa cells (Figure 5B). Apilimod-driven TGFβ receptor internalization was further confirmed in a cell-surface labelling of TGFβ receptors. Indeed, Apilimod significantly reduced the cell-surface binding of fluorescent TGFβ, both in primary cardiac fibroblasts (Figure 5C) and HeLa cells (Figure 5D), indicating a reduction in the amount of surface available receptors in Apilimod-treated cells. Interestingly, Apilimod did not alter the localization of the tyrosine-kinase EGF receptor, the G-coupled receptor P2Y12 or of the adhesion molecule ICAM1 (Figure S2), suggesting that Apilimod targets specifically the TGFβ receptor localization.

Apilimod has been shown to specifically inhibit the lipid kinase PIKfyve 15, 19. This inhibitory effect was validated in our *in vitro* (Figure S3A) and *in vivo* models (Figure S3B) by monitoring the amount of PIKfyve products PI5P and PI(3,5)P_2_. To further confirm the implication of PIKfyve, we used mouse embryonic fibroblasts (MEF) isolated from WT or hypomorphic PIKfyve^β-geo/β-geo^ mice 14. As shown in Figure 5E, TGFβ-R2 was restricted to the plasma membrane in resting DMSO-treated PIKfyve^+/+^ MEF, while Apilimod induced the intracellular sequestration of TGFβ-R2, as observed in cardiac fibroblasts and HeLa cells. Similarly, in MEF isolated from PIKfyve^β-geo/β-geo^ mice, TGFβ-R2 was absent from the cell surface and essentially localized in intracellular vesicles (Figure 5E). The implication of PIKfyve was also confirmed by the use of a structurally distinct inhibitor, the YM201636 compound 12, which induced TGFβ-R2 internalization in treated cells, similarly to Apilimod-treatment (Figure 5F). Moreover, overexpression of PIKfyve^WT^ ablated the effect of both Apilimod and YM201636 on TGFβ-R2 localization (Figure 5F). This strongly suggests that Apilimod effect on TGFβ-R2 localization is due to its specific inhibition of PIKfyve.

**Figure 6:**
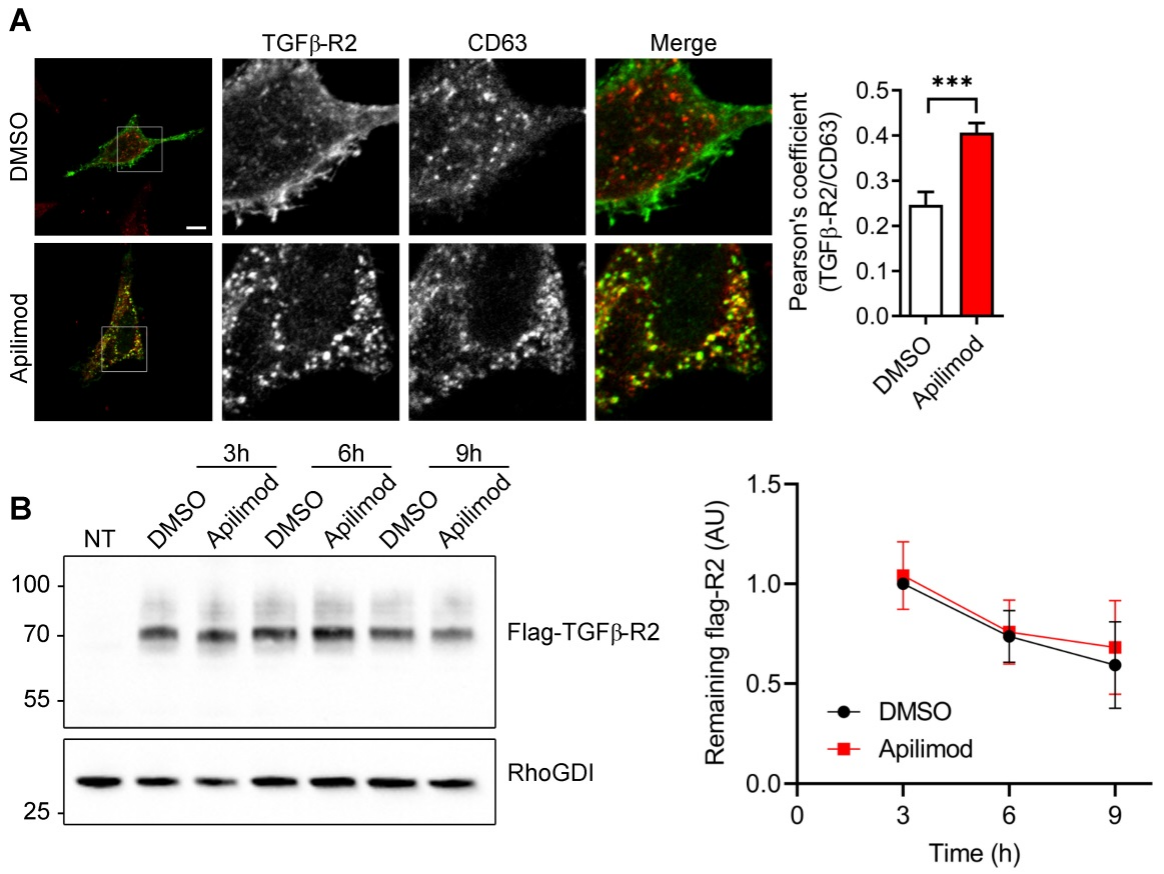
** Apilimod induces TGF-βR2 mislocalization to late-endosomes. (A)** Serum-starved HeLa cells expressing flag-TGFβ-R2 were treated as indicated with Apilimod or DMSO, fixed and stained with an anti-flag antibody (green) and an anti-CD63 antibody (red). Bar is 10 µm. Right panel shows the quantification of colocalization. n = 17-37 cells across 3 independent experiments. Student's t-test (unpaired, two-tailed) ***p < 0.001.** (B)** Non-transfected (NT) or HEK cells expressing flag-TGFβ-R2 were serum-starved and treated with DMSO or Apilimod for the indicated times. Cell lysates were blotted with an anti-flag antibody to assess the amount of remaining flag-TGFβ-R2. RhoGDI was used as a loading control. n = 3 independent experiments.

Next, we sought to identify the subcellular compartment in which TGFβ-R2 receptor is internalized upon Apilimod treatment. As shown in Figure S4A, Apilimod provoked an extensive vacuolation of EEA1-endosomes, but did not induce the internalization of TGFβ-R2 in this compartment. Similarly, Apilimod did not induce accumulation of TGFβ-R2 in caveolae (Figure S4B). In comparison, Apilimod increased significantly the colocalization of TGFβ-R2 with the late endosomal marker CD63 (Figure 6A), indicating that PIKfyve inhibition triggered the sequestration of TGFβ-R2 in a CD63-positive late endosomal compartment. Interestingly, we found that Apilimod did not alter the half-life of TGFβ-R2 (Figure 6B), suggesting that internalized TGFβ-R2 was not targeted for lysosomal degradation and that PIKfyve is more likely to be implicated in the receptor recycling back to the plasma membrane.

### Apilimod dampens TGFβ signaling pathway

**Figure 7:**
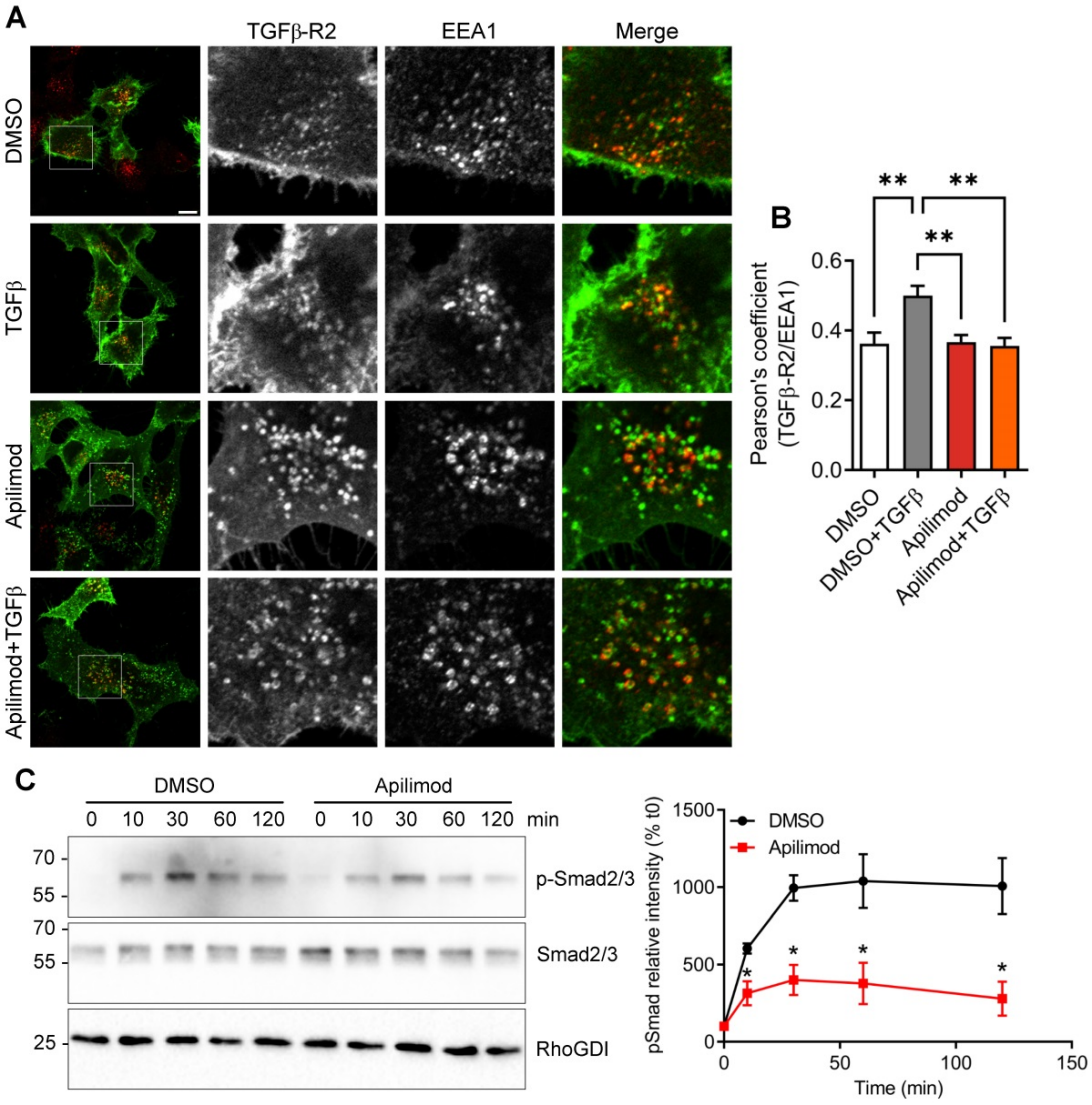
** Apilimod prevents TGFβ downstream signaling. (A)** Serum-starved HeLa cells expressing flag-TGFβ-R2 were treated with Apilimod or DMSO as indicated, stimulated with TGFβ, fixed and stained with anti-flag (green) and anti-EEA1 (red) antibodies. Bar is 10 µm. **(B)** Quantification of colocalization between TGFβ-R2 and EEA1. n = 16-37 cells across 3 independent experiments **(C)** Primary cardiac fibroblasts were serum-starved and pre-treated with DMSO or Apilimod, before stimulation with TGFβ for the indicated time. Cell lysates were blotted for phosphorylated Smad2/3 (p-Smad2/3), total Smad2/3, or RhoGDI as a loading control. Quantification if shown on the right panel. n = 3 independent experiments. ANOVA followed by Bonferroni's post-hoc test or Student's t-test (unpaired, two-tailed), **p < 0.01.

Upon binding to its ligand, TGFβ receptor is known to be first internalized to EEA1-positive endosomal compartment, mediating the downstream signaling pathway 8. Indeed, in DMSO-treated cells, TGFβ stimulation induced the internalization of the receptor in EEA1-positive vesicles (Figure 7A-B). In contrast, this ligand-mediated internalization was lost in Apilimod-treated cells (Figure 7A-B). Furthermore, upon TGFβ stimulation, a fraction of the receptor is internalized in CD-63-positive late endosomes (Figure S5). Interestingly, in Apilimod-treated cells challenged with TGFβ, we did not observe a cumulative effect on the colocalization of TGFβ-R2 and CD63 (Figure S5), indicating that the internalized receptor is not responsive to TGFβ stimulation. Consequently, we found that Apilimod reduced TGFβ-dependent phosphorylation of the canonical downstream effectors Smad2/3 in cardiac fibroblasts (Figure 7C). Collectively, these results show that PIKfyve controls TGFβ receptor trafficking and regulates its downstream signaling.

The TGFβ signaling pathway is a common feature among several fibrotic pathologies such as pulmonary, renal or hepatic diseases 31. Therefore, we wondered whether long-term Apilimod treatment might affect other tissues beside the heart. We then performed qPCR analysis to address the hepatic fibrotic and inflammatory status Apilimod-treated mice subjected to TAC. As shown in Figure S6, 4 weeks TAC did not increase the hepatic fibrosis status (as demonstrated by the expression levels of collagen 1 and TGFβ), nor the inflammatory status of the liver (as shown by IL-6, TNF-α, F4/80 and CCL2 expression levels). Moreover, in sham animals, Apilimod treatment had no consequences on the hepatic fibrotic or inflammatory status (Figure S6), showing that systemic administration of Apilimod has no consequences on other tissues.

### Apilimod controls TGFβ receptor localization *in vivo*

**Figure 8:**
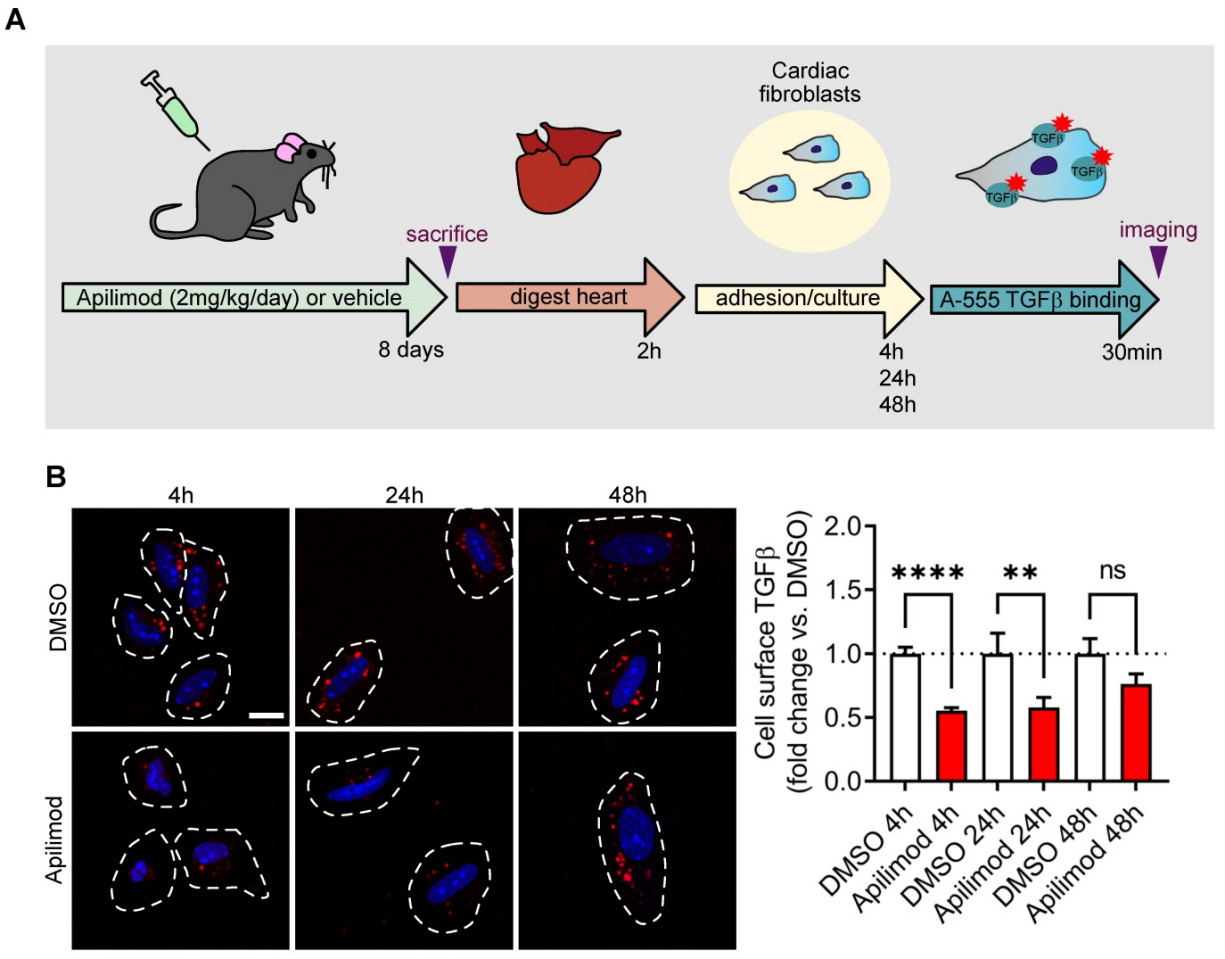
** Apilimod induces TGFβ receptor internalization *in vivo*. (A)** Schematic of the experimental setting. **(B)** Cardiac fibroblasts isolated from vehicle or Apilimod-treated mice were plated and processed for cell surface binding of fluorescent TGFβ (TGFβ-A555, shown in red) after the indicated time in culture. Nuclei were stained with DAPI (shown in blue). Bar is 10 µm. Quantification is shown on the right from 34-265 cells from 3 technical replicates from 2-4 mice per group. ANOVA followed by Bonferroni's post-hoc test, **p < 0.01; ****p < 0.0001; ns, non-significant.

Lastly, in order to investigate whether Apilimod controls TGFβ receptor localization *in vivo*, mice were treated with the inhibitor for 8 consecutive days, and cardiac fibroblasts were isolated and let to adhere on a gelatine matrix. We then used the cell-surface labelling of TGFβ receptors in order to investigate the amount of available receptors. As shown in Figure 8, in cardiac fibroblasts isolated from DMSO-treated mice, we observed a punctate labelling, similar to what was observed *in vitro* (see Figure 5C-D). Strikingly, in cardiac fibroblast isolated from Apilimod-treated mice, a dramatic reduction in cell surface TGFβ labelling was observed (Figure 8B), suggesting that PIKfyve inhibition induced TGFβ receptor internalization *in vivo*. Interestingly, this inhibition was lost upon establishment of the culture (i.e. after 48 h), indicating that the effect of Apilimod on TGFβ receptor is reversible.

Altogether, our results demonstrate for the first time that Apilimod controls TGFβ receptor trafficking and its subsequent signaling pathway and that Apilimod treatment opens the way to new anti-fibrotic therapeutic strategies.

## Discussion

Fibrotic remodeling is a common pathological hallmark in numerous acute and chronic diseases leading to organ injury and failure. Activation of naive fibroblasts and their transition to myofibroblasts is an adaptative and necessary phenomenon in the short term but highly deleterious if prolonged, resulting in irreversible scar formation, organ stiffness and failure 2, 31. In this context, we show that pharmacological inhibition of PIKfyve by Apilimod limits myocardial fibrosis development upon injury, culminating in the preservation of organ function. *In vitro*, our work demonstrates that Apilimod dampens TGFβ-mediated cell migration and invasion and fibroblast activation. Importantly, at the molecular level, we revealed that Apilimod induces the accumulation of TGFβ-R2 in intracellular vesicles of late endosomal nature. Given the fact that TGFβ-R2 continuously recycles between plasma membrane and endosomes 30, it is tempting to postulate that PIKfyve could be involved in the control of this recycling process. We propose that PIKfyve inhibition dramatically alters endosomal sorting of the TGFβ-R2, therefore re-routing it from the recycling pathway to the degradative pathway. However, Apilimod did not induce TGFβ-R2 degradation, which can be explained by the defect in lysosomal acidification typically observed upon PIKfyve-inhibition by the less-potent inhibitor YM201636 32-34. Interestingly, it has been shown in podocytes that PIKfyve knock-out induces an arrest of trafficking of the recycling pathway, causing extensive accumulation of early, late and recycling endosomes, namely EEA1, Rab5 and Rab11 vesicles 35. Consistently, it has been shown that PIKfyve inhibition by YM201636 blocks the recycling of the tight junction adhesion molecules Claudin-1 and Claudin-2 in epithelial cells 36, and that PIKfyve regulates the Rab11-dependent recycling of two potassium channels 37. Alternatively, one might hypothesize that PIKfyve inhibition triggers TGFβ-R2 internalization from the plasma membrane. Although this cannot be ruled out, such an early function for PIKfyve would be unprecedented, as the enzyme is typically involved in late endosomal maturation. Moreover, whether PIKfyve inhibition alters the dimerization of the TGFβ receptors R1 and R2, their phosphorylated or ubiquitinated status, remain to be addressed in future work. Interestingly, it has been shown that PIKfyve inhibition by YM201636 or siRNA-mediated knock-down does not alter EGF receptor internalization 38, 39, which is consistent with our results. However, this has been challenged by several studies 12, 40, 41. In any case, all these studies refer to ligand-induced receptor internalization. Here, however, we show that PIKfyve inhibition alters the steady-state localization of TGFβ-R2, independently from any ligand. Altogether, these findings suggest that PIKfyve functions in the control of the endosomal system are diverse and receptor-specific.

The lipid kinase PIKfyve produces the two signaling lipids PI(3,5)P_2_ and PI5P. Although the role of PI(3,5)P_2_ in the endosomal maturation has been extensively documented 14, 42-44, the role of endogenous PI5P is scarcely described 45, 46. We previously demonstrated that tampering with PI5P by artificially increasing its levels altered the trafficking and steady-state localization of different cell-surface proteins, like the EGF receptor 22, 47 and the adhesion molecule ICAM1 18. This is reminiscent of what we observe here on TGFβ-R2, with the internalization of the receptor in the absence of its ligand, although the situation is quite different. Indeed, the pharmacological inhibition of PIKfyve would certainly decrease the levels of PI5P. This suggests that a tightly controlled balance in PIKfyve activity is needed to ensure a proper trafficking of these receptors through the endosomal system.

TGFβ receptors internalization is known to play either a positive or a negative role in downstream signaling 8. Our results show that the vacuolar retention of TGFβ-R2 induced by PIKfyve inhibition is in favour of a negative regulation of the downstream signaling pathway. Indeed, we found that Apilimod induced the TGFβ-R2 retention in late endosomes, a non-signaling compartment as opposed to early/signaling endosomes. Moreover, we show that Apilimod reduces the phosphorylation of Smad2/3, crucial downstream effectors of the TGFβ signaling pathway. Finally, Apilimod reduces TGFβ response, ultimately leading to a reduction in the expression of TGFβ pro-fibrotic and pro-inflammatory target genes.

Although TGFβ is the main route for fibroblast activation, the contribution of other routes, like inflammatory factors, cannot be ruled out. Apilimod has been shown to possess strong anti-inflammatory properties 15, 48, 49. Consistently, we found in our study that Apilimod reduces the production of several key pro-inflammatory cytokines and the infiltration of inflammatory macrophages within the myocardium. This is reminiscent of what we observed in a diabetic cardiomyopathy mouse model, although we found that Apilimod treatment failed to reduce systemic inflammation 17. The specific contribution of both pathways (pro-fibrotic vs pro-inflammatory) is difficult to address, particularly *in vivo*. Although we clearly demonstrate here that PIKfyve plays a direct role on TGFβ response in cardiac fibroblasts, we cannot rule out an effect of Apilimod on other cardiac cell types. Indeed, we found previously 17 and as demonstrated in this study that Apilimod administration reduces cardiomyocyte hypertrophic response. Interestingly, we show here that Apilimod administration does not completely abrogate the hypertrophic response in TAC mice. Lastly, although we previously showed that Apilimod acts directly on cardiomyocytes, we cannot rule out the existence of a paracrine signaling from fibroblasts on myocytes, particularly important *in vivo*.

TGFβ triggers a canonical signaling pathway involved in many organs, regulating both physiological and pathological states 31. In our study, although we observed a potent effect of Apilimod on the pathological heart, we found that long-term treatment does not affect the hepatic fibrotic or inflammatory status. This is consistent with the fact that Apilimod is well tolerated in human, as observed during the first phases of several clinical trials 50, 51. Clinically, Apilimod possesses a real advantage as the oral formulation provides a clear improvement over injectable therapies. Although tested in two different clinical trials, against Crohn's disease and rheumatoid arthritis 50, 51, it failed to demonstrate an effect above placebo, suggesting that systemic administration is not potent to decrease the overall inflammation inherent to these pathologies. Moreover, TGFβ signaling dysregulation is often observed in several cancers, mostly linked to fibrosis development and cancer-associated fibroblasts 4. Recently, Apilimod has been shown to possess anticancer activity against B-cell non-Hodgkin lymphoma 52 and is currently tested in a clinical trial, and different inhibitors of PIKfyve are promising anticancer drugs 53.

It has been shown that PIKfyve is required for the entry of several pathogen bacteria and viruses, including Ebola and Marburg viruses 54, 55 and Legionella pneumophila 56. More recently, PIKfyve has been implicated in the entry of the SARS-CoV-2, responsible for the newly emerged pandemic zoonosis 57. Of note, Apilimod was one of the first hit in a large-scale compound repurposing screen, demonstrating strong antiviral efficacy 58. Although previous studies for PIKfyve function in these processes were centred on its role on phagocytosis and pathogen clearing 56, the recent work from Ou and colleagues suggests that PIKfyve could regulate the recycling of angiotensin convertase enzyme 2 (ACE2), the cell surface receptor of the virus 57. While the authors did not provide direct evidence that PIKfyve controls ACE2 recycling, their data strongly suggest this is the case, which is reminiscent of what we observe in our study for the TGFβ receptor.

## Conclusions

In conclusion, we demonstrate that Apilimod dampens cardiac fibrotic activation, and propose this compound as a regulator of TGFβ signaling. Therefore, PIKfyve pharmacological inhibition by Apilimod may provide new strategies to control fibrotic remodeling associated with chronic diseases.

## Supplementary Material

Supplementary figures and tables.

## Figures and Tables

**Table 1:** Echocardiograhic parameters of mice.

	Sham	Sham+Apilimod	TAC	TAC+Apilimod
EF (%)	64.33±1.05	62.17±4.03	40.00±8.07**	59.13±3.29$
SF (%)	34.67±0.88	33.35±2.92	19.80±4.21**	32.29±2.56$
LVPWd (mm)	0.83±0.61	0.89±0.07	1.23±0.03***	0.99±0.05$
IVSd (mm)	0.78±0.05	0.88±0.04	1.22±0.02****	1.09±0.02$,§§§§
LV mass (mg)	112.3±5.7	118.5±5.2	206.6±16.3****	167.3±10.4$,§§

EF: ejection fraction; SF: shortening fraction; LVPWd: end-diastolic ventricular wall thickness; IVSd: end-diastolic intraventricular septum thickness; LV mass: left ventricle mass. ANOVA followed by Bonferroni's post-hoc test: Sham vs. TAC **p < 0.01, ***p < 0.001, ****p < 0.0001; TAC vs. TAC+Apilimod ^$^p < 0.05; Sham vs. TAC+Apilimod ^§§^p < 0.01, ^§§§§^p < 0.0001.

## Abbreviations

TGFβ: transforming growth factor beta
PIKfyve: phosphoinositide kinase, FYVE-type zinc finger containing
ECM: extracellular matrix
SMA: smooth muscular actin
IL: interleukin
ROS: reactive oxygen species
Smad: Mothers Against Decapentaplegic Homolog
TAC: transverse aortic constriction
CTGF: connective tissue growth factor
TNF-α: tumour necrosis factor-alpha
ANP: natriuretic peptide A
BNP: brain natriuretic peptide
β-MHC: β-myosin heavy chain
CCL2: C-C motif chemokine ligand 2
mRNA: messenger ribonucleic acid
DMSO: dimethyl sulfoxide
PBS: phosphate buffered saline
WGA: wheat germ agglutinin
HBSS: Hank's Balanced Salt Solution
BSA: bovine serum albumin
DMEM: Dulbecco's Modified Eagle Medium
FBS: fetal bovine serum
PFA: paraformaldehyde
RIPA buffer: Radioimmunoprecipitation assay
ECL: electrochemiluminescence
qRT-PCR: Quantitative Polymerase Chain Reaction
ANOVA: analysis of variance
MMP9: matrix metalloprotease 9
EGF: epidermal growth factor
ICAM1: intercellular adhesion molecule 1
MEF: mouse embryonic fibroblast
HEK: human embryonic kidney
EEA1: early endosome antigen 1
PI(3,5)P_2_: Phosphatidylinositol 3,5-bisphosphate
PI5P: Phosphatidylinositol 5-phosphate
ACE2: angiotensin I converting enzyme 2
