# Supplementary material for: Apilimod alters TGFβ signaling pathway and prevents cardiac fibrotic remodeling
Source: Theranostics. 2021 Apr 19;11(13):6491–506. doi: 10.7150/thno.55821 (PMC8120213; doi:10.7150/thno.55821)

SUPPLEMENTAL TABLE AND FIGURES

Supplementary Table S1. qRT-PCR primers used in this study

Genes	Primer Sequence (forward)	Primer Sequence (reverse)
Gapdh	5'-TGGCAAAGTGGAGATTGTTGCC-3'	5'-AAGATGGTGATGGGCTTCCCG-3'
Rplpo	5'-TGACATCGTCTTTAAACCCCG-3'	5'-TGTCTGCTCCCACAATGAAG-3'
Hprt	5'-TGAAAGACTTGCTCGAGATGTCAT-3'	5'-TCCAGCAGGTCAGCAAAGAA-3'
Rsp29	5'-GTCTGATCCGCAAATACGGG-3'	5'-AGCCTATGTCCATCGCGTACT-3'
β-Mhc	5'-AGGGCGACCTCAACGAGAT-3'	5'-CAGCAGACTCTGGAGGCTCT-3'
α-Sma	5'-GTCCCAGACATCAGGGAGTAA-3'	5'-TCGGATACTTCAGCGTCAGGA-3'
Bnp	5'-GACAGCTCTTGAAGGACC-3'	5'-GTACCGATCCGGTCTAT-3'
Anp	5'-ATCACCCTGGGCTTCTTC-3'	5'-CCTCCTTGGCTGTTATCTT-3'
Col1 (heart)	5'-CCTGCCTGCTTCGTGTAA-3'	5'-GGTCACGTTCAGTTGGTCA-3'
Col1 (liver)	5'-TGCTTTCTGCCCGGAAG-3'	5'-GGGATGCCATCTCGTCCA-3'
Col3	5'-TCCTGAAGATGTCCTTGATGTACAG-3'	5'-TTCAGAGACTTCTTTACATTGCCATT-3'
Fibronectin 1	5'-GGTGTAGCACAACTTCCAATTACG-3'	5'-GGAATTTCCGCCTCGAGTCT-3'
α-skeletal actin	5'-TACCACCGGCATCGTGTTG-3'	5'-CCAGGTCCAGACGCATGAT-3'
Ctgf	5'-CTCCACCCGAGTTACCAATG-3'	5'-TGGCGATTTTAGGTGTCCG-3'
Periostin	5'-AAGAGATGGTCACTTCACGC-3'	5'-GCACTGGAGGGTATTTAGGATG-3'
Il-6 (heart)	5'-CAAAGCCAGAGTCCTTCAGAG-3'	5'-GTCCTTAGCCACTCCTTCTG-3'
Il-6 (liver)	5'-GCCCACCAAGAACGATAGTCA-3'	5'-CAAGAAGGCAACTGGATGGAA-3'
Ccl2	5'-GCAGTTAACGCCCCACTCA-3'	5'-CCAGCCTACTCATTGGGATCA-3'
F4/80	5'-TGACAACCAGACGGCTTGTG-3'	5'-GCAGGCGAGGAAAAGATAGTGT-3'
Tnf-α (heart)	5'-CTTCTGTCTACTGAACTTCGGG-3'	5'-CAGGCTTGTCACTCGAATTTTG-3'
Tnf-α (liver)	5'-TGGGACAGTGACCTGGACTGT-3'	5'-TTCGGAAAGCCCATTTGAGT-3'
Tgfβ	5'-GAGCCCGAAGCGGACTACTA-3'	5'-CACTGCTTCCCGAATGTCTGA-3'
Mmp9	5'-TGAGTCCGGCAGACAATCCT-3'	5'-CGCCCTGGATCTCAGCAATA-3'

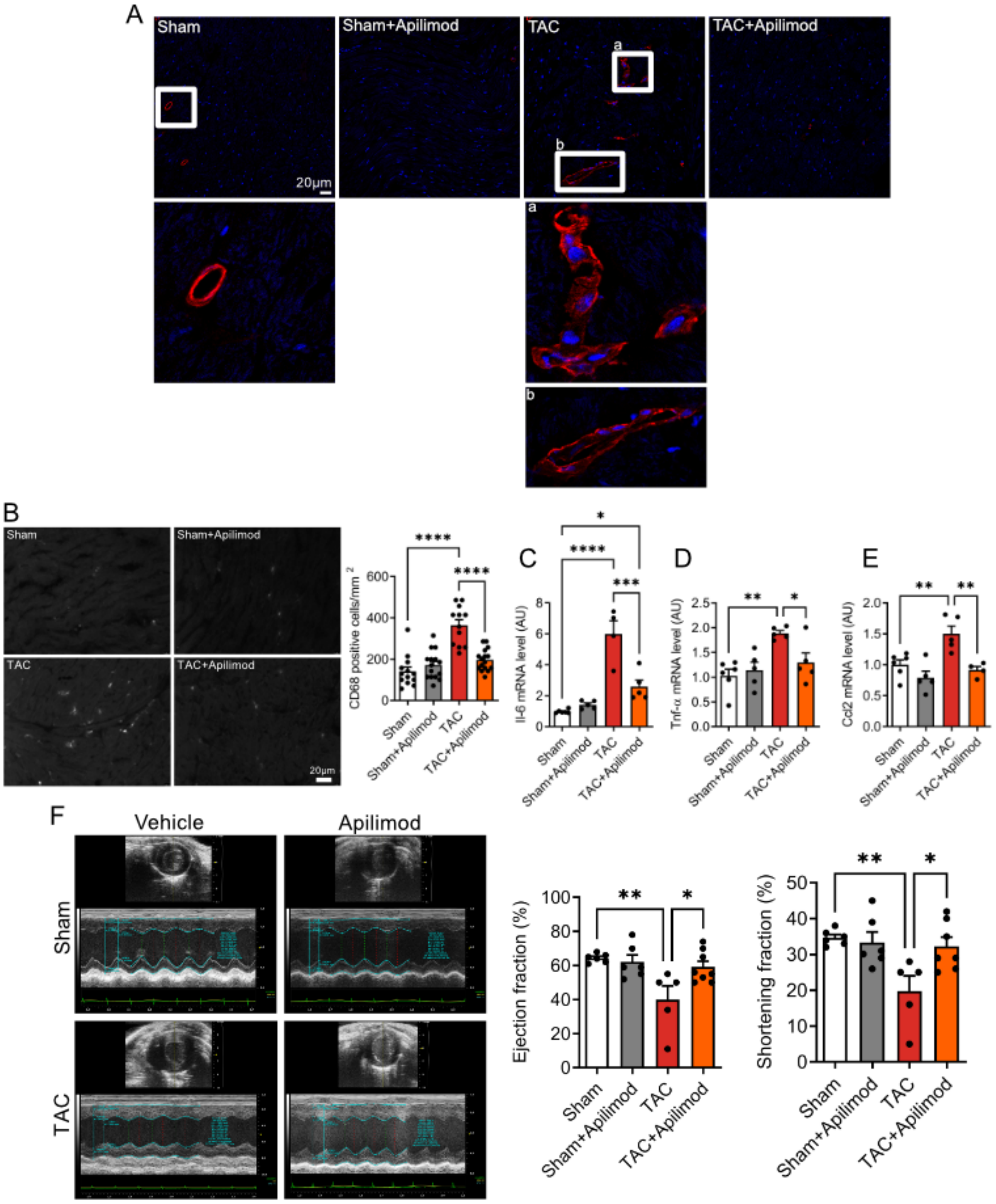

Figure S1. α-SMA and myocardial inflammation are reduced in Apilimod-treated TAC-mice.

(A) α−SMA immunostaining (shown in red) of cardiac cryosections from treated mice. Nuclei were stained with DAPI (in blue). Enlarged panels show typical images for blood vessels (Sham), or highlight single activated cardiac fibroblasts (panels a and b, TAC). **(B)** CD-68 immunostaining and quantification of CD-68 positive cells density in cardiac cryosections of treated mice. Quantification was performed from 3-4 images across 3-5 mice per group. **(C-E)** Expression levels of Il-6, Tnf-α and Ccl2 in cardiac tissues by qRT-PCR. n = 4-6 mice per group. **(F)** Representative 2D-M-mode echocardiography images of sham- or TAC-operated mice treated with Apilimod and echocardiographic measurements of ejection fraction and shortening fraction. n = 5-7 mice per group. ANOVA followed by Bonferroni's post-hoc test, *p < 0.05; **p < 0.01; ***p < 0.001; ****p < 0.0001.

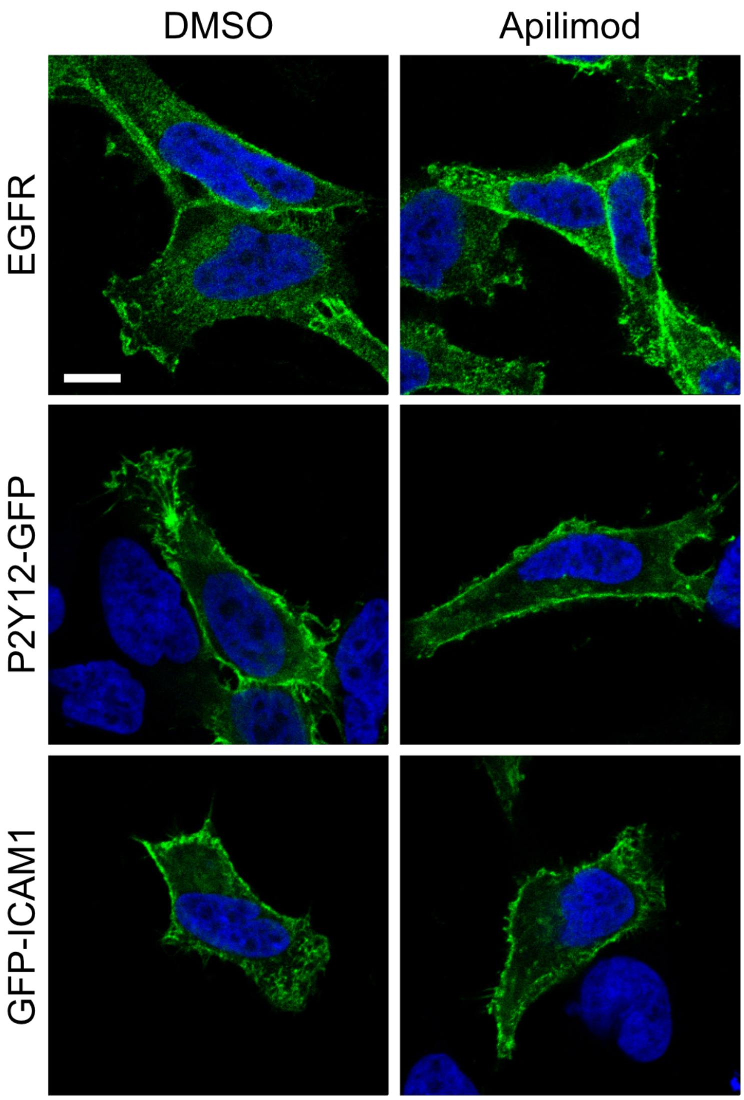

Figure S2. Apilimod does not alter EGFR, P2Y12 and ICAM1 steady-state localization. **(A)** HeLa cells were serum-starved and treated with Apilimod or vehicle only (DMSO), fixed and stained for endogenous EGFR or overexpressed P2Y12-GFP or GFP-ICAM1 (shown in green). Nuclei were stained with DAPI (in blue). Bar is 10 µm.

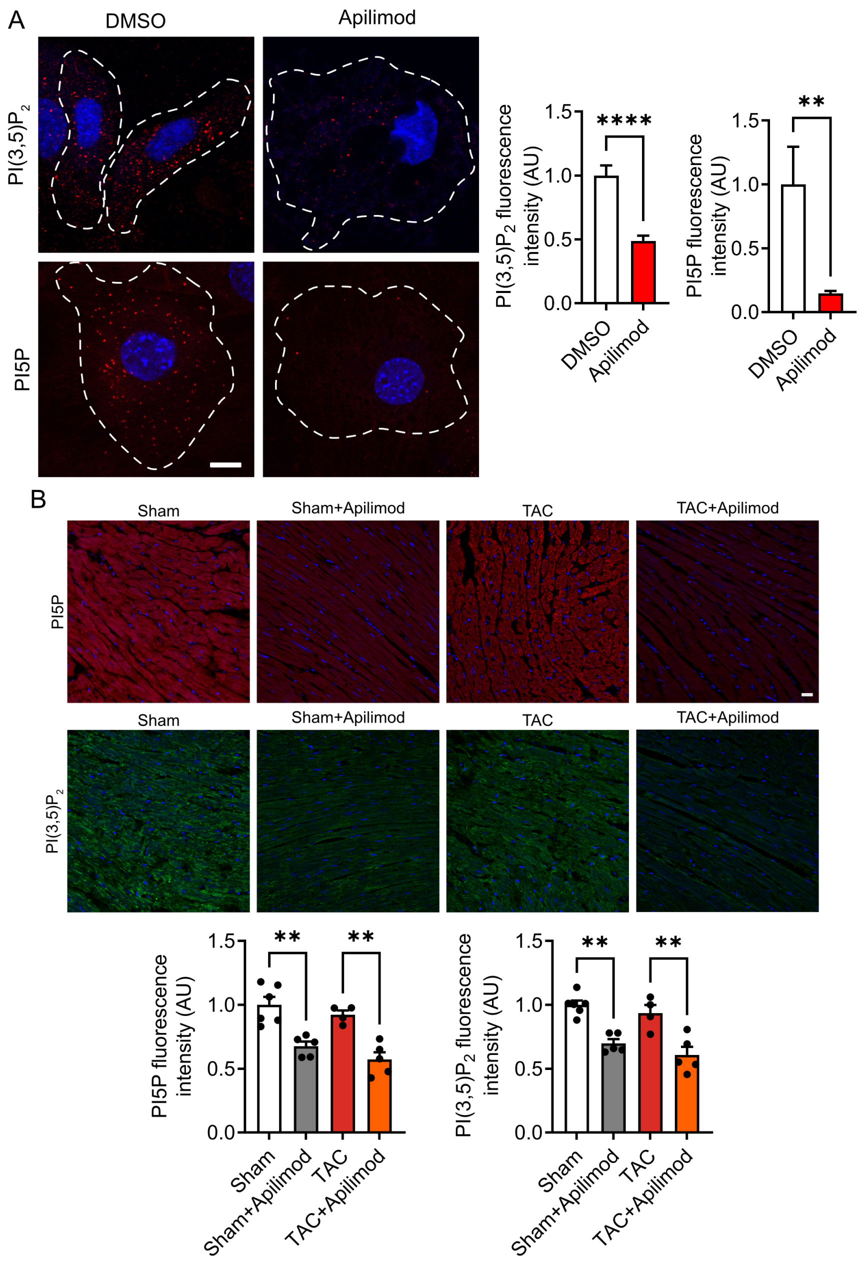

Figure S3. Apilimod inhibits PIKfyve activity in vivo and in vitro.

(A) Primary cardiac fibroblasts were treated with Apilimod (100 nM) or with DMSO alone for 48 h, fixed and stained for PI5P or PI(3,5)P_2 (shown in red). Nuclei were stained with DAPI (in blue). Bar is 10 µm. Quantifications are shown on the right, n = 9-16 cells. Student's t-test (unpaired, two-tailed), $^{**}p < 0.01$; $^{****}p < 0.0001$.

(B) Cardiac cryosections from mice treated as indicated were immunostained for PI5P (shown in red) or PI(3,5)P2 (shown in green). Nuclei were stained with DAPI (in blue). Bar is 20 µm. Quantifications shown on the right are from 4-6 mice per group. ANOVA followed by Bonferroni's post-hoc test, $^{**}p < 0.01$.

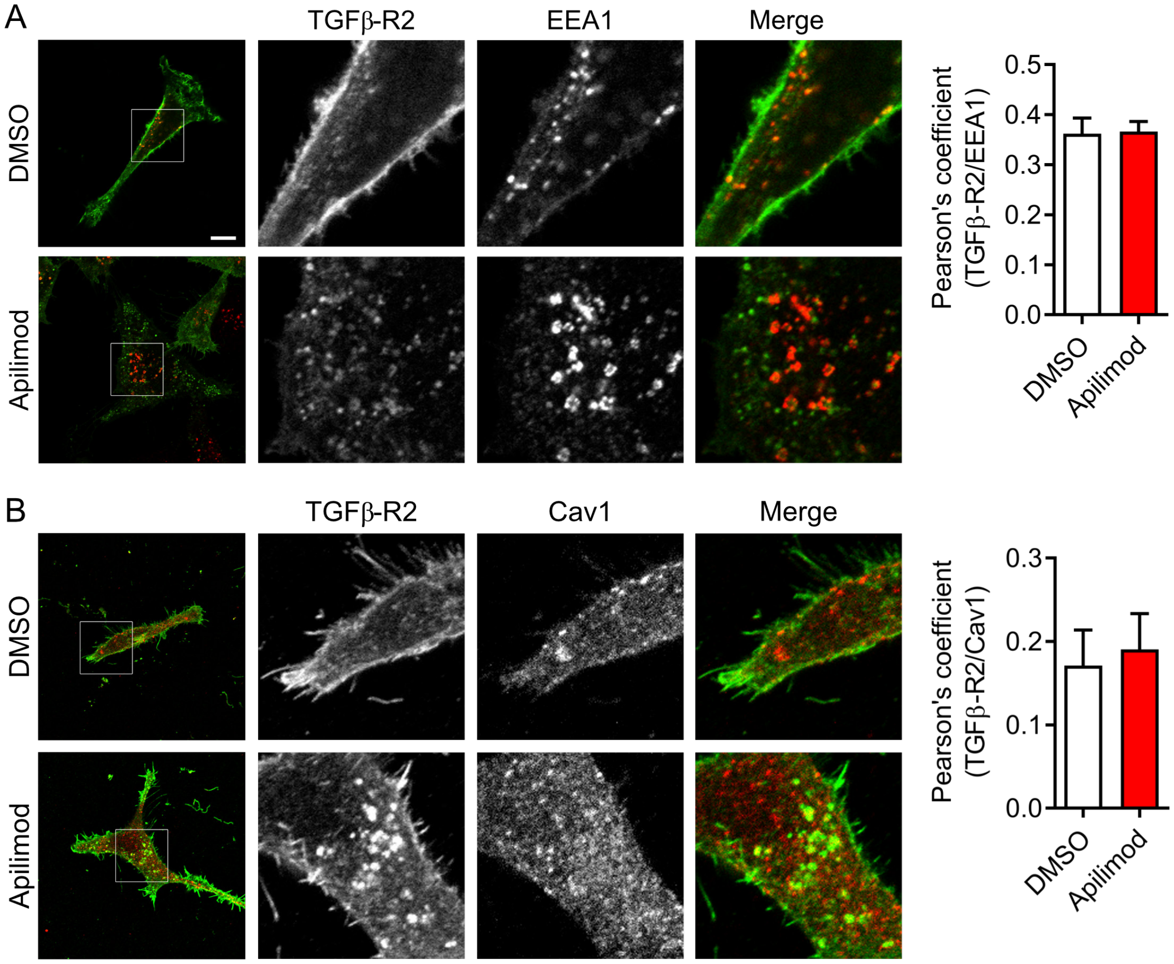

Figure S4. Internalized TGFβ-R2 does not colocalized with EEA1 or Cav1 in Apilimod-treated cells.

(A-B) Serum-starved HeLa cells expressing flag-TGFβ-R2 were treated with Apilimod or vehicle only (DMSO), fixed and stained with an anti-flag antibody (green) and anti-EEA1 (A) or anti-Cav1 (B) antibodies (red). Bar is 10 µm. Right panels show the quantification of colocalization between flag-TGFβ-R2 and the indicated markers.

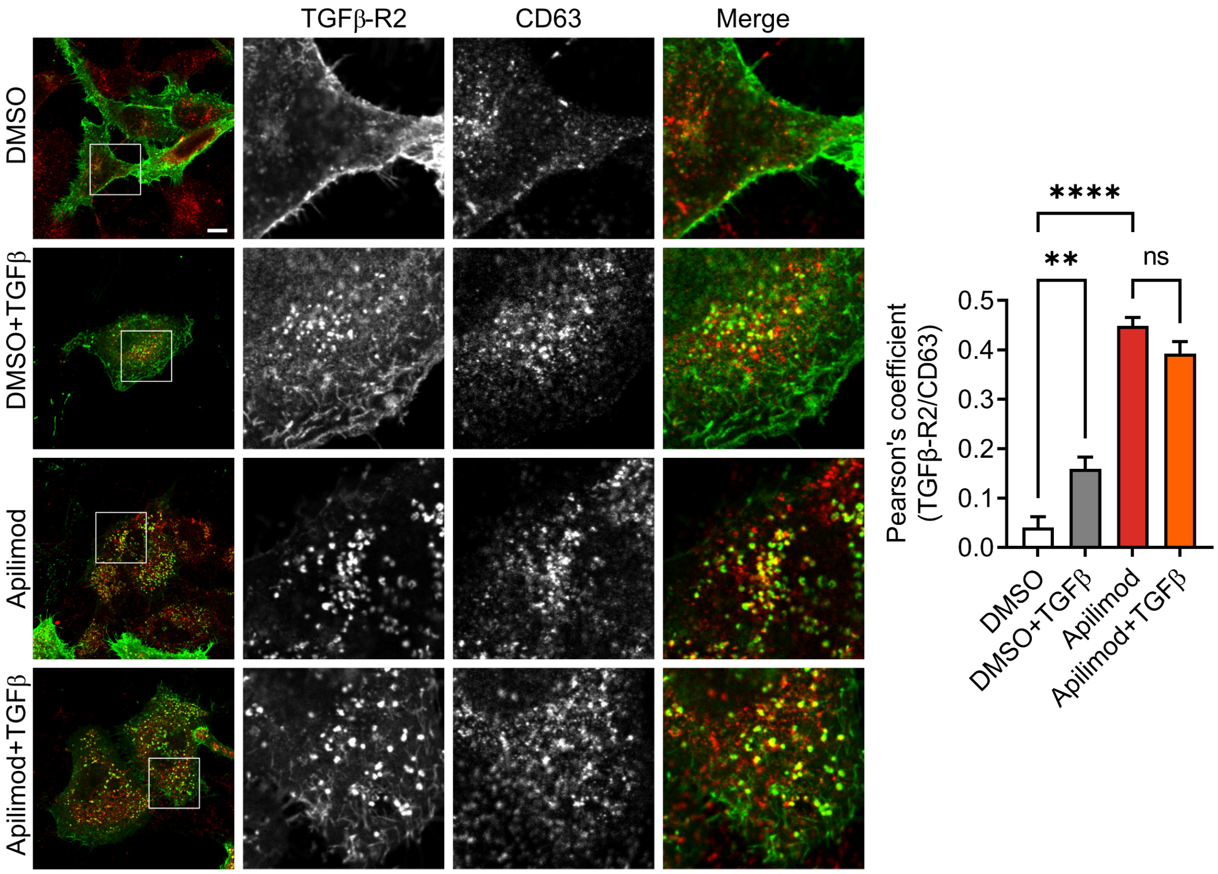

Figure S5. TGFβ-R2 colocalization with CD63 in Apilimod and TGFβ treated cells. Serum-starved HeLa cells expressing flag-TGFβ-R2 were treated with Apilimod or DMSO as indicated, stimulated with TGFβ, fixed and stained with anti-flag (in green) and anti-CD63 (in red) antibodies. Bar is 10 µm. Quantification of colocalization between TGFβ-R2 and CD63 is shown on the right. n = 36-53 cells across 3 independent experiments. ANOVA followed by Bonferroni's post-hoc test, $^{**}p < 0.01$; $^{****}p < 0.0001$; ns, non-significant.

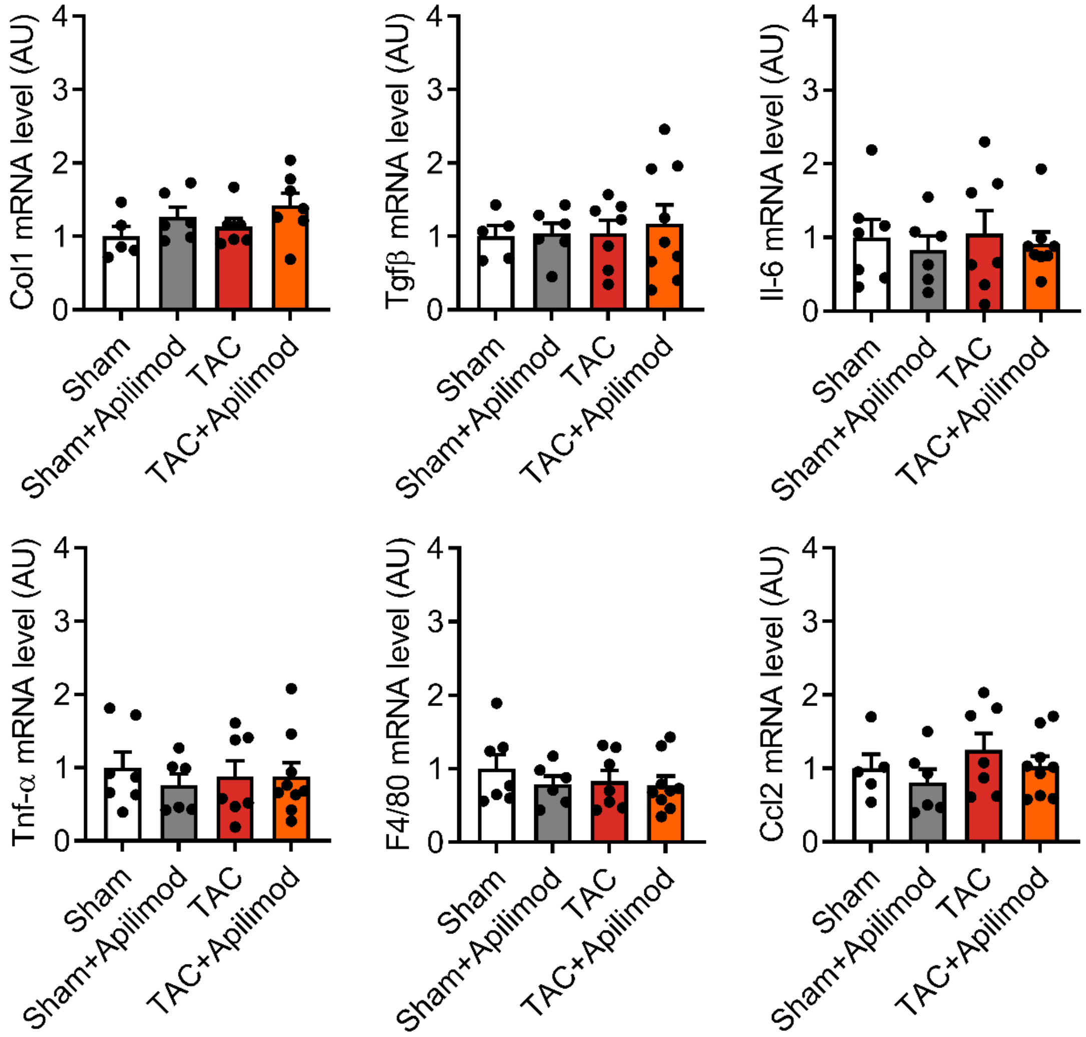

Figure S6. Fibrotic and inflammatory status of mice liver.

Expression levels of collagen 1 (Col1), Tgfβ, Il-6, Tnf-α, F4/80 and Ccl2 were measured by qRT-PCR in hepatic tissues of mice treated as indicated. n = 5-9 mice per group.

FULL UNEDITED GELS

Full unedited gel for Figure 2G

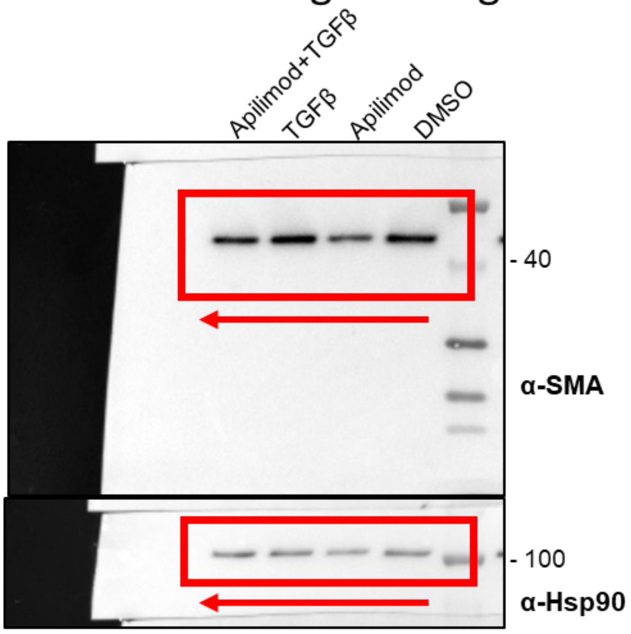

Full unedited gel for Figure 5B

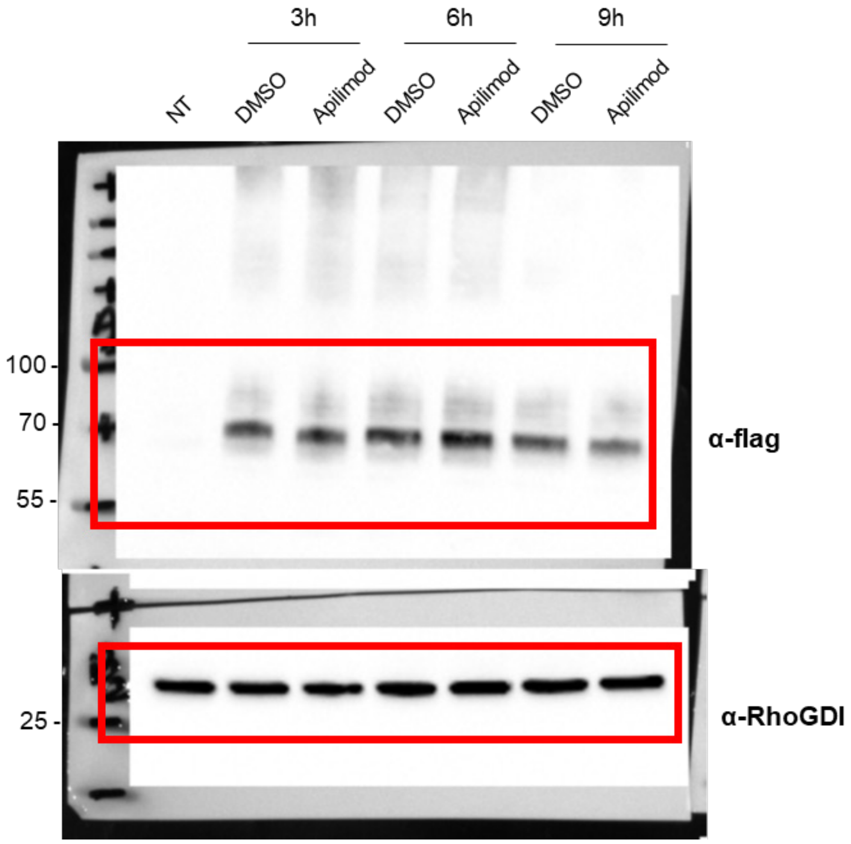

Full unedited gel for Figure 6C

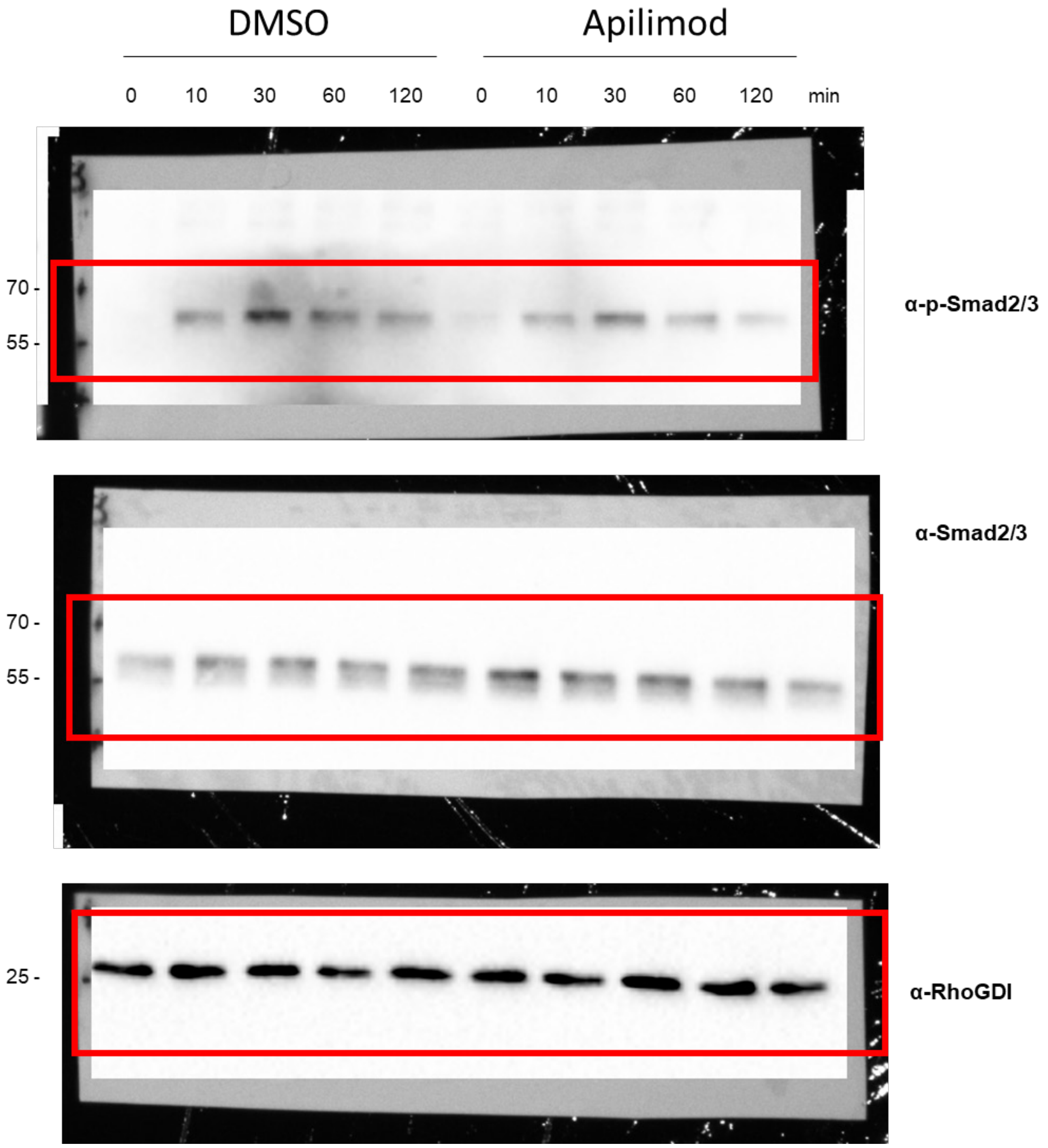

Supplement: Supplementary file 1 — Supplementary figures and tables. [file thnov11p6491s1.pdf]
